# Atom-by-atom assembly reveals structure–performance control in PdCu catalysts for CO_2_ hydrogenation to methanol

**DOI:** 10.1039/d5sc06681f

**Published:** 2025-10-20

**Authors:** Louise R. Smith, Emerson C. Kohlrausch, Kieran J. Aggett, Mario Samperi, Sadegh Ghaderzadeh, Andreas Weilhard, Luke T. Norman, Isla E. Gow, Yifan Chen, Giuseppe Bonura, Catia Cannilla, Elena Besley, David J. Morgan, Thomas J. A. Slater, Andrei N. Khlobystov, Jesum Alves Fernandes, Graham J. Hutchings

**Affiliations:** a Max Planck-Cardiff Centre on the Fundamentals of Heterogeneous Catalysis FUNCAT, Translational Research Hub, Cardiff University Maindy Road Cardiff CF24 4HQ UK hutch@cardiff.ac.uk; b School of Chemistry, University of Nottingham, University Park Nottingham NG7 2RD UK jesum.alvesfernandes@nottingham.ac.uk; c CNR-ITAE Istituto di Tecnologie Avanzate per l’Energia Nicola Giordano Via Comunale S. Lucia 5 Messina 98126 Italy

## Abstract

The catalytic conversion of CO_2_ to methanol using bimetallic materials presents a promising pathway for sustainable chemical production. A major challenge is the lack of atomic-level control over the catalyst structure and composition, which hinders the understanding of each metal's role in activity and selectivity. Here, we present a solvent-free on-surface assembly of PdCu bimetallic particles, directly from atoms, on ZnO with precise control of the order and quantity of metal atoms added. This atomic-defined interface reveals when atoms are added simultaneously, the metal with stronger ZnO binding governs particle size, but when introduced sequentially the first metal determines particle size. The simultaneously deposited PdCu exhibits the highest reported methanol productivity for PdCu-systems, achieving 8.2 mol h^−1^ mol_metal_^−1^ at 270 °C and 20 bar. In this catalyst, Cu enhances CO_2_ adsorption, suppresses Zn incorporation into the PdCu structure and modulates Pd binding strength to reaction intermediates. This enhances methanol selectivity while maintaining high Pd-driven CO_2_ conversion.

## Abstract 2

Atomically controlled PdCu catalysts assembled directly from metal atoms on ZnO uncover how deposition order governs particle structure and activity, delivering exceptional CO_2_-tomethanol productivity and selectivity.

## Graphical abstract

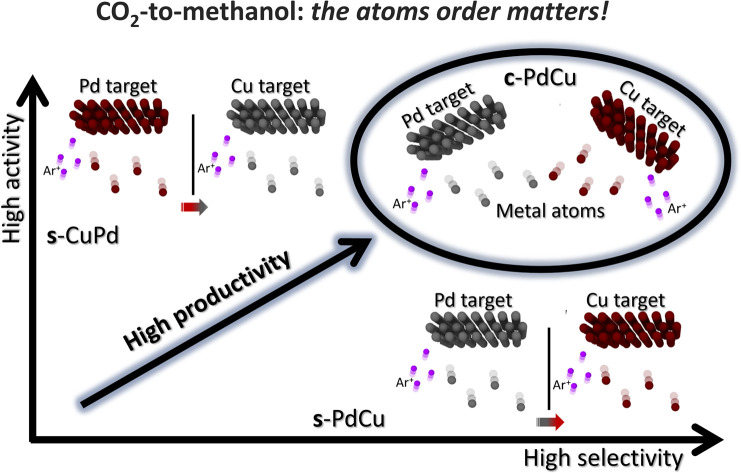

## Introduction

Industrial methanol synthesis from syngas (CO + 2H_2_ ⇄ CH_3_OH) is well established using copper based catalysts, mainly focusing on Cu/ZnO/Al_2_O_3_ (CZA) catalysts.^1–3^ Whilst CZA has also been widely explored for methanol synthesis from CO_2_, these catalysts typically exhibit high activity for the reverse water gas shift reaction (RWGS; CO_2_ + H_2_ ⇄ CO + H_2_O), lowering methanol yields.^4,5^ Extensive research has been carried out in the literature to understand the catalytic activity of CZA for CO_2_ to methanol, including the cause of catalyst deactivation.^6^ Malte Behrens *et al.* highlighted a significant understanding of the relationship between Cu and ZnO in the industrial CZA catalyst, whereby the increased catalytic activity is a result of the higher proportion of defective Cu sites and partial coverage of the Cu particles surface with ZnO. These factors strengthen the binding of intermediates and therefore lower the energy barriers for methanol production.^7^ Although copper-based catalysts are widely studied, their catalytic performance still limited due to deactivation.^8^ As water is produced alongside methanol from CO_2_ feeds, steam causes significant sintering of both Cu and ZnO in CZA catalysts, resulting in catalyst deactivation.^9^ Consequently, a range of catalytic materials have been investigated for the conversion of CO_2_ to methanol, including ZnZrO_*x*_ materials,^10–13^ supported Pd catalysts,^14–17^ and In_2_O_3_-based materials,^18–21^ among others.^22–25^

Alloying Pd and Cu has been shown to be particularly effective for the hydrogenation of CO_2_ to methanol, as demonstrated by Jiang *et al.*^26–29^ for silica-supported bimetallic PdCu catalysts. The ratio of metals plays a crucial role, as the methanol space time yield (STY) was found to be highest with a Pd/(Pd + Cu) atomic ratio of 0.34; at lower Pd ratios, high selectivity to CO resulted in lower methanol yields, whilst lower CO_2_ conversions were observed at higher Pd ratios.^26,27^ DFT calculations showed that the activation barrier for the formation of the formate intermediate for a range of Pd/(Pd + Cu) ratios correlated well with methanol STY.^28^ The effect of support on the performance of PdCu catalysts has been investigated, with methanol production following the trend: ZrO_2_ ≈ TiO_2_ > Al_2_O_3_ > CeO_2_ > SiO_2_.^30^ The superior activity of the ZrO_2_ and TiO_2_ supported catalysts was attributed to the moderate strength of metal-support interaction, with the higher degree of metal-support interaction present in the Pd–Cu/CeO_2_ resulting in restructuring of the Pd–Cu alloy and reducing catalytic performance. Importantly, Dorado and co-workers reported the use of trimetallic Pd–Cu–Zn/SiC catalysts for ambient pressure CO_2_ hydrogenation.^31^ A higher methanol formation rate was obtained over the trimetallic Pd–Cu–Zn catalyst compared with the bimetallic Pd–Cu, Pd–Zn, and Cu–Zn analogues, while the precise origins of the synergistic effect in the trimetallic catalyst are not well understood.

The examples above clearly demonstrate that CO_2_-to-methanol conversion depends critically on the interplay between nanoparticle composition, structure, and support interactions. However, achieving precise atomic-level control over these factors remains a significant challenge. Overcoming this requires constructing nanoparticles atom by atom, in a defined sequence and ratio, to systematically tailor the active sites and metal-support interfaces that govern both methanol selectivity and CO_2_ conversion efficiency.

In this study, we construct bimetallic PdCu on zinc oxide support by depositing metal atoms in different sequences and ratios by magnetron sputtering for CO_2_-to-methanol conversion. The metal atom deposition mode, whether sequentially depositing Cu then Pd (s-CuPd), Pd then Cu (s-PdCu), co-depositing Pd and Cu (c-PdCu) or physically mixing Pd/ZnO and Cu/ZnO, was found to be crucial for catalytic activity. The c-PdCu mode demonstrated enhanced synergy between the metals, which significantly improved CO_2_ hydrogenation. Further exploration of the activity enhancement was achieved by varying the Pd/Cu ratio of the c-PdCu, resulting in a catalyst that outperforms all other PdCu catalysts systems in productivity for methanol synthesis. Comparative data with similar CO_2_ hydrogenation systems (Table S3) confirm its superior performance.

## Results

### PdCu/ZnO catalysts

**Fig. 1:**
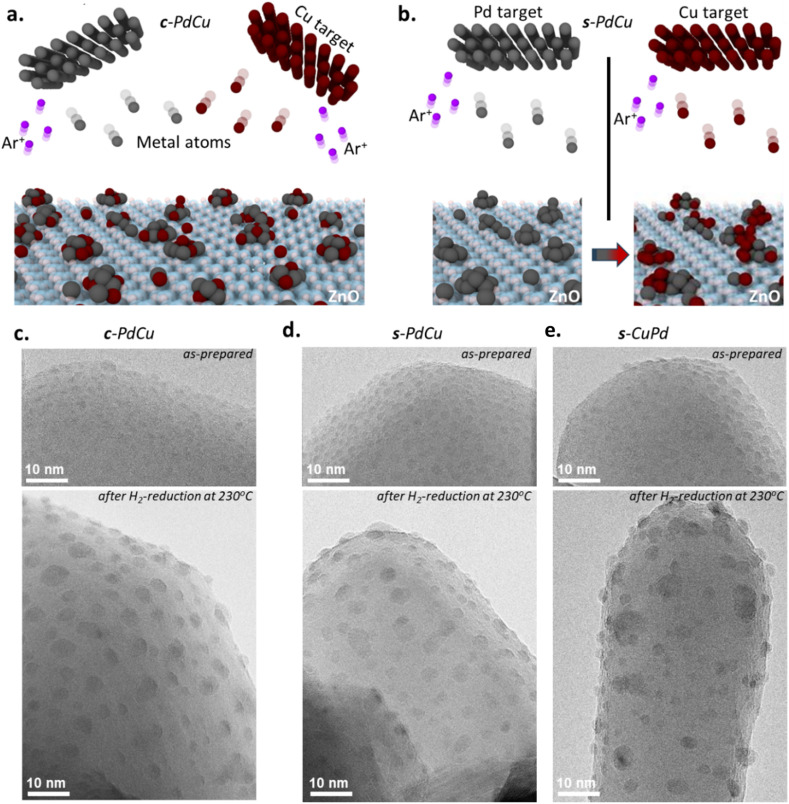
Bimetallic catalyst assembly by two modes of deposition of Pd and Cu atoms onto ZnO support: (a) illustration of simultaneous Pd and Cu deposition metal deposition used to prepare c-PdCu; (b) illustration of sequential Pd and Cu deposition metal deposition used to prepare s-PdCu or s-CuPd, depending on the order; (c–e) TEM images illustrating the distribution of metal particle dispersion on ZnO support for (c) c-PdCu, (d) s-PdCu and (e) s-CuPd.

Cu and Pd possess distinct physico-chemical properties that can significantly influence the self-assembly of bimetallic clusters, thereby determining their final morphology and composition, and consequently, their catalytic performance. To systematically investigate these effects on CO_2_ hydrogenation to methanol, we fabricated PdCu catalysts supported on ZnO by varying the deposition sequence of Cu and Pd atoms, without using chemical surfactants or ligands using a magnetron sputtering approach (Fig. 1).^32–35^ Although this technique has been widely employed in thin-film applications,^36^ its use in the preparation of supported metal nanoparticles, particularly CO_2_ hydrogenation, remains relatively rare.^37–39^ For example, *Romeggio et al.* prepared low-surface-area NiGa thin films with limited relevance to nanoparticles catalysts,^39^ while *Rossi et al.* employed monometallic catalysts selective for CO production.^40^

In the magnetron sputtering approach used here, a flow of metal atoms is directed onto the ZnO support, where the individual atoms self-assemble into particles, guided exclusively by their surface properties such as diffusion barriers and the density of surface defects (Fig. 1).^35,41,42^ Since the same support (ZnO) was used throughout this work, this approach enabled a direct correlation between the intrinsic properties of the metals and their resulting catalytic performance. Two deposition modes were employed (co-deposition and sequential deposition), to make three different catalysts: c-PdCu, in which Pd and Cu atoms were co-deposited at the same time; s-PdCu, in which Pd atoms were deposited first, followed by Cu; and then s-CuPd, where the sequence was reversed, leading to three different bimetallic structures (Fig. 1). All catalysts were assembled with similar Pd : Cu atomic ratios, as verified by inductively coupled plasma optical emission spectroscopy (ICP-OES). The catalysts were characterised both in their as-prepared state and after reduction at 230 °C under hydrogen, which represents the state of the catalyst immediately before the reaction.^43–46^

High resolution transmission electron microscopy (HRTEM) analysis of c-PdCu, s-PdCu, and s-CuPd before and after reduction revealed nanoparticles with flattened pyramidal shapes, well distributed across the (0001) and (010-1) facets of ZnO (Fig. 1). The c-PdCu and s-PdCu particles both exhibited average diameters of 2.0 ± 0.5 nm, while the average diameter of the s-CuPd particles was slightly larger at 2.3 ± 0.5 nm. This trend persisted after the reduction step at 230 °C, conducted immediately prior to the reaction, with particle sizes increasing slightly to 3.0 ± 0.8 nm for both c-PdCu and s-PdCu, and more substantially to 4.1 ± 1.5 nm for s-CuPd (Fig. 1c and d). Notably, the average particle size and distribution were significantly larger for s-CuPd compared to s-PdCu. This observation suggests that, in sequential deposition, the metal deposited first dictates the size of the resulting bimetallic clusters, as it preferentially occupies the primary binding sites and initiates nucleation before the second metal is deposited. To further investigate this phenomenon, we performed density functional theory (DFT) calculations of Pd and Cu binding energies on the ZnO (0001) facet (Fig. S1). The results indicate that Pd binds more strongly to ZnO than Cu, with binding energies of −4.25 eV and −2.96 eV, respectively. Interestingly, both Pd and Cu atoms preferentially bind to zinc atoms rather than to oxygen atoms within the ZnO structure. To emulate the initial stages of cluster formation, a second Pd or Cu atom was deposited on ZnO surface. The calculated Pd–Pd/ZnO and Cu–Cu/ZnO binding energies were −3.13 eV and −3.31 eV, respectively. These results show that Cu–Cu/ZnO bonding is 0.4 eV more favourable than Cu binding to the ZnO support, whereas Pd binding to ZnO support is 0.12 eV more favourable than Pd–Pd/ZnO bonding. These findings confirm that Pd consistently exhibits a stronger interaction with the ZnO support than Cu. This stronger Pd–ZnO binding limits Pd atom migration on the ZnO surface, leading to the formation of smaller Pd particles compared to Cu. This trend is reflected in the observed morphology of the corresponding bimetallic clusters (Fig. 1d and e). Hence, our findings support the conclusion that in sequential deposition, the particle size largely reflects that of the metal deposited first. In contrast, in co-deposition, the final particle size is primarily governed by the metal with the stronger binding affinity to the support, Pd in this case. Importantly, these behaviours can only manifest under conditions where metal-support interactions are not masked or altered by ligands or surfactants, underscoring the intrinsic nature of the observed effects.

**Fig. 2:**
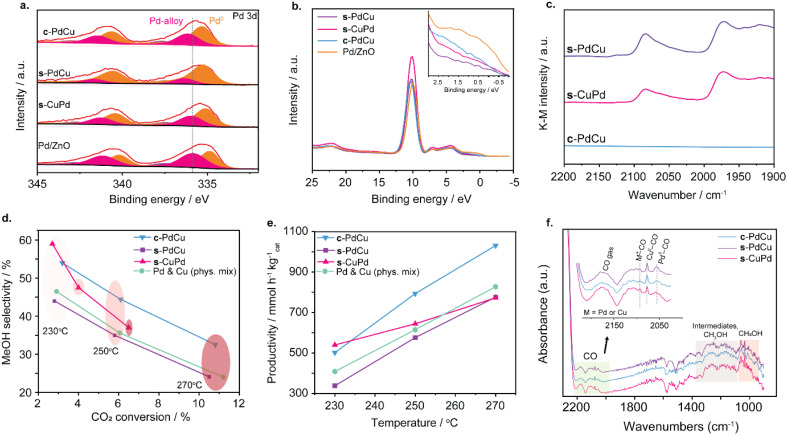
PdCu/ZnO catalysts prepared by different metal deposition modes. (a) XPS Pd 3d spectra for PdCu/ZnO catalysts post-reduction with Pd/ZnO also shown for reference; (b) valence band spectra for PdCu/ZnO catalysts post-reduction with the ZnO support shown for reference; (c) CO-DRIFTS spectra for PdCu/ZnO catalysts obtained under flowing N_2_; (d) MeOH selectivity *vs.* CO_2_ conversion for c-PdCu, s-PdCu and s-CuPd and a physical mixture of Pd/ZnO and Cu/ZnO; (e) MeOH productivity as a function of reaction temperature for c-PdCu, s-PdCu and s-CuPd and a physical mixture of Pd/ZnO and Cu/ZnO. Catalysts were reduced at 230 °C (1 hour, 5 °C min^−1^) prior to characterisation and testing; (f) DRIFT spectra of PdCu/ZnO catalysts under reaction conditions (20 bar, 270 °C, GHSV = 6400 NL kg_cat_^−1^ h^−1^), showing the CO_2_ intermediates and methanol regions. An inset of the CO region highlights the CO at gas phase, M^+^ – CO, Cu^0^–CO and Pd^0^–CO vibrations (M = Pd or Cu).

X-ray photoelectron spectroscopy (XPS) of the Pd, Cu and PdCu catalysts on ZnO was conducted both before (Fig. S2–S3a and Table S1) and after reduction (Fig. 2a, S3b, Table S1, and Fig. S4, S5). The Pd 3d XPS spectra show a PdO peak at 337.0 eV prior to reduction, which disappears after reduction, resulting in the presence of only peaks at 335.0 eV and 336.2 eV corresponding to Pd^0^ and a Pd-alloy, respectively, regardless of the deposition mode. Notably, s-PdCu exhibits a higher ratio of metallic Pd after reduction compared to the other bimetallic catalysts (Fig. 2a). The Pd^0^ peak for monometallic Pd/ZnO appears at a lower binding energy of 335.0 eV than that of Pd^0^ for c-PdCu, s-CuPd and s-PdCu, which all appear at a similar binding energy of 335.5 eV. This indicates charge transfer from Pd to Cu caused by the flow of electrons from the fully filled Pd 4d orbital to the partially-filled Cu 4s orbital, confirming the interaction between Pd and Cu alloy.^47–53^ The Cu 2p XPS spectra show the presence of both metallic Cu at 932.0 eV and CuO at 933.5 eV before and after reduction (Fig. S3). After reduction, a 0.4–0.5 eV downward shift in energy is observed, along with a substantial increase in the proportion of Cu^0^. The valence band spectra (Fig. 2b) are dominated by ZnO, but nevertheless, there appears to be a shift away from the Fermi level for PdCu *vs.* Pd, indicative of charge transfer from Pd to Cu,^47,53^ and further highlighting the interaction between Pd and Cu in the bimetallic catalysts.

To further investigate the final structure of PdCu/ZnO, we calculated the relative surface concentrations of Pd and Cu for all catalysts (Table S1). s-CuPd and s-PdCu exhibited similar surface concentrations of Pd and Cu (*ca.* 2.2%), whereas a significantly higher surface concentration (*ca.* 4.3%) was observed for c-PdCu, despite all catalysts having comparable total metal loadings. These findings indicate that Pd and Cu are more randomly distributed for c-PdCu compared to the sequentially deposited samples, supporting the self-assembly mechanism proposed in Fig. 1.

Diffuse Reflectance Infrared Fourier Transform Spectroscopy (CO-DRIFTS) revealed a difference in bonding CO with bimetallic centres, depending on whether Pd and Cu were simultaneously or sequentially deposited (Fig. 2c). In the presence of flowing CO (Fig. S6), all spectra were dominated by Pd–CO adsorption with bands at *ca.* 2094 cm^−1^ and 1978 cm^−1^, representing linearly bound and bridge-bound CO on Pd^0^, respectively.^54,55^ Following CO adsorption, spectra were taken under flowing N_2_ to remove gas phase CO molecules (Fig. 2c). Bands at 2085 and 1972 cm^−1^ corresponding to bonded CO, remained for both s-CuPd and s-PdCu, with a minor adsorption band visible at 2126 cm^−1^ representing CO bound to Cu.^56^ However, under the same conditions, no CO bands were visible for c-PdCu, suggesting significantly weaker interactions between CO and any Pd atoms in c-PdCu (Fig. 2c). This corroborates with a stronger charge transfer from Pd to Cu observed in the XPS of c-PdCu, which may weaken the binding strength of adsorbed CO to Pd,^49,57^ once again suggesting a greater degree of interaction between Pd and Cu on ZnO surfaces when the atoms are simultaneously deposited.

Temperature-programmed desorption (CO_2_-TPD) was carried out to assess the CO_2_ adsorption behaviour of the catalysts (Fig. S7). All samples exhibit broadly similar desorption profiles; however, quantitative analysis reveals notable differences in CO_2_ uptake. The integrated CO_2_-TPD signals correspond to10 mL g^−1^ for c-PdCu, 13 mL g^−1^ for s-PdCu, and 4 mL g^−1^ for s-CuPd. These results demonstrate that Cu plays a critical role in enhancing CO_2_ adsorption. When Pd is deposited last (s-CuPd), thus covering the Cu particles, the CO_2_ adsorption capacity decreases significantly.

The catalytic performance of PdCu/ZnO catalysts prepared *via* different metal deposition modes is shown in Fig. 2d and e. A physical mixture of Pd/ZnO (1 wt%) and Cu/ZnO (1 wt%) was included for comparison. A detailed investigation of the effect of the reduction temperature at 230 *vs.* 400 °C and loading at 0.5 and 1 wt% of Pd/ZnO and Cu/ZnO are shown in Fig. S8–S11. To ensure comparable metal loadings to the bimetallic catalysts, the individual components were pelleted, and 0.25 g of each was combined and loaded into the reactor. Across the tested temperature range, the s-PdCu and the physical mixture exhibited similar CO_2_ conversions and methanol selectivities, leading to comparable methanol productivities. The reverse sequence catalyst, s-CuPd, showed comparable CO_2_ conversion to the other samples at 230 °C, but with higher methanol selectivity, resulting in increased methanol productivity at this temperature. However, at 250 °C and 270 °C, s-CuPd displayed lower CO_2_ conversion with higher selectivity, leading to the comparable methanol productivity at 250 °C and 270 °C with s-PdCu and the physical mixture. In contrast, the c-PdCu catalyst achieved CO_2_ conversions similar to s-PdCu and the physical mixture but consistently higher methanol selectivity. As a result, c-PdCu delivered methanol productivities comparable to s-CuPd at 230 °C, and significantly higher than all other catalysts at 250 °C and 270 °C with values of 794 and 1031 mmol h^−1^ kg_cat_^−1^, respectively, corresponding to 6.3 and 8.2 mol h^−1^ mol_metal_^−1^. Importantly, HR-TEM images of c-PdCu, s-PdCu, and s-CuPd catalysts show no significant changes in mean particle diameter or size distribution after catalysis (Fig. S12).

*In situ* DRIFT experiments were performed on c-PdCu, s-PdCu, and s-CuPd catalysts under reaction conditions (20 bar, 270 °C) to elucidate the superior performance of c-PdCu over the other bimetallic systems (Fig. 2f and S13, S14). Prior to introducing the reaction mixture (CO_2_/H_2_/N_2_ = 23/69/8 mol mol^−1^) into the reactor, all bimetallic catalysts were reduced under a 5% H_2_/Ar flow at 230 °C for 1 hour. Across all catalysts, bands at 1003 cm^−1^, 1032 cm^−1^, and 1060 cm^−1^ were detected, corresponding to gas-phase methanol. In the 1300–1700 cm^−1^ region, features ascribed to surface-bound carbonate and bicarbonate intermediates were observed for all catalysts.^25,58^ The broad and poorly resolved nature of these signals is likely due to overlapping contributions from water vapour. The pronounced bands in s-PdCu may indicate strong stabilisation of formate- and carbonate-like intermediates on its surface.

The 1950–2200 cm^−1^ region, characteristic of CO stretching vibrations, provides insight into the oxidation state and CO adsorption modes of Cu and Pd. A peak at 2077 cm^−1^, assigned to linearly adsorbed CO on metallic Cu, was observed for all three catalysts. Additionally, a small peak at 2094 cm^−1^ was present in all bimetallic systems, which can be attributed to CO adsorbed on Pd^*δ*+^ or Cu^*δ*+^. Similar peak has been reported for CuZn-based catalysts, where charge transfer from Cu to Zn leads to Cu^*δ*+^ species.^59–61^ Based on our spectroscopy results in Fig. 2a–c, which indicated charge transfer from Pd to Cu, this feature was assigned to CO bounded to Pd^*δ*+^ sites.

Interestingly, a distinct peak at 2057 cm^−1^ corresponding to CO adsorbed on metallic Pd was observed, with a significantly highest intensity for s-PdCu. This observation is consistent with XPS results (Fig. 2a), which revealed a higher proportion of metallic Pd in s-PdCu compared to s-CuPd and c-PdCu. These findings demonstrate that s-PdCu exhibits stronger binding to reaction intermediates, such as CO and formate-like species, relative to s-CuPd and c-PdCu. Notably, s-PdCu displayed the lowest methanol selectivity among the tested catalysts, whereas s-CuPd and c-PdCu demonstrated comparable selectivity at 270 °C (Fig. 2d). In contrast, s-CuPd exhibited the lowest CO_2_ conversion, while s-PdCu and c-PdCu achieved similar, significantly higher conversions than s-CuPd under identical conditions (Fig. 2d). These findings clearly highlight that Cu plays a dominant role in tuning methanol selectivity, whereas Pd is the primary contributor to CO_2_ conversion. Therefore, the order of metal deposition strongly influences the catalytic performance. When Cu is deposited first, as in s-CuPd, the resulting catalyst exhibits enhanced selectivity towards methanol, albeit at the expense of CO_2_ conversion. Conversely, when Pd is deposited first, as in s-PdCu, the catalyst displays high activity but lower methanol selectivity due to insufficient electronic modulation of Pd by Cu. In the case of c-PdCu, where Pd and Cu are intimately mixed at the atomic level, both favourable Cu-induced modulation of intermediate binding strength and high Pd-driven activity are simultaneously achieved (Fig. 2d and e). These synergistic properties are central to the superior catalytic performance of c-PdCu for CO_2_-to-methanol conversion.

**Fig. 3:**
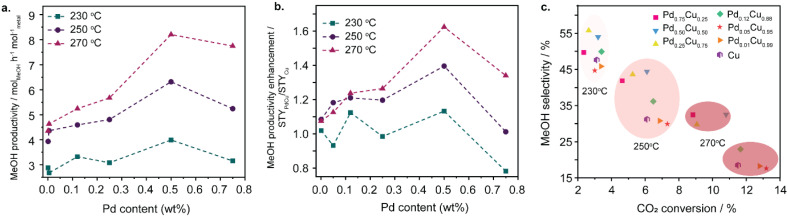
Performance of PdCu/ZnO catalysts with a variety of Pd : Cu molar ratios and fixed total loadings of 1 wt%. (a) MeOH productivity; (b) enhancement in MeOH productivity for Pd_*x*_Cu_*y*_ catalysts *vs.* monometallic Cu; (c) MeOH selectivity *vs.* CO_2_ conversion.

To further investigate the c-PdCu catalyst, we prepared a series of PdCu/ZnO catalysts with varying Pd : Cu ratios. The total metal loading was fixed at 1 wt%, with compositions ranging from Pd_0.01_Cu_0.99_ to Pd_0.75_Cu_0.25_. Chemical and electronic properties were examined by XPS (Fig. S15–S18), which revealed no significant differences compared to c-Pd_0.50_Cu_0.50_, with peaks assigned to Pd^0^ and Pd alloy. In contrast, no peak attributed to alloyed Pd was observed for Pd_0.12_Cu_0.88_, Pd_0.05_Cu_0.95_ and Pd_0.01_Cu_0.99_, although this may be due to the low amounts of Pd present in these samples (<0.12 wt% Pd). The Cu 2p spectra showed the presence of Cu^0^ and CuO for all samples. The catalytic performance of the c-PdCu catalysts with varying compositions is presented in Fig. 3.

Methanol productivity as a function of Pd concentration is shown in Fig. 3a, with the data normalised to moles of metal. A volcano plot was observed with a maximum methanol productivity achieved over Pd_0.50_Cu_0.50_ at all reaction temperatures, with values of 4.0, 6.3 and 8.2 mol h^−1^ mol_metal_^−1^ at 230, 250 and 270 °C, respectively. Fig. 3b shows the enhancement in methanol productivity for all c-Pd_*x*_Cu_*y*_ catalysts, compared with Cu/ZnO, where all catalysts have a fixed loading of 1 wt%. Consistent with Fig. 3a, a clear maximum was again observed at a Pd_0.5_Cu_0.5_.

In terms of methanol selectivity, catalysts with higher Pd content, Pd_0.75_Cu_0.25_, Pd_0.50_Cu_0.50_, Pd_0.25_Cu_0.75_, exhibited enhanced selectivity across the full temperature range (Fig. 3c). Interestingly, negligible CH_4_ formation was detected over Pd_0.50_Cu_0.50_, Pd_0.25_Cu_0.75_, and even Pd_0.12_Cu_0.88_ (<0.1%). Although Pd_0.12_Cu_0.88_ showed similar methanol selectivity to Pd_0.50_Cu_0.50_ and Pd_0.25_Cu_0.75_ at 250 °C, its selectivity dropped at higher temperatures. In contrast, Pd-rich (Pd_0.75_Cu_0.25_) and Cu-rich (Pd_0.05_Cu_0.95_ and Pd_0.01_Cu_0.99_) catalysts exhibited significantly higher CH_4_ formation, with selectivities from 0.8 to 0.5% for Pd_0.75_Cu_0.25_, 0.5 to 0.2% for Pd_0.05_Cu_0.95_, and 0.3 to 0.1% for Pd_0.01_Cu_0.99_ (Table S2). These are critical results for catalyst application at large scales, as even at low levels methane formation is detrimental to methanol synthesis. Unlike CO, which can be recycled through subsequent hydrogenation, CH_4_ must be purged from the system, leading to increased operational costs.^62^ Methane has been reported as a by-product in methanol synthesis from CO_2_ over Cu/ZnO catalysts with a range of Cu oxidation states,^43^ as well as monometallic Pd catalysts.^63^

**Fig. 4:**
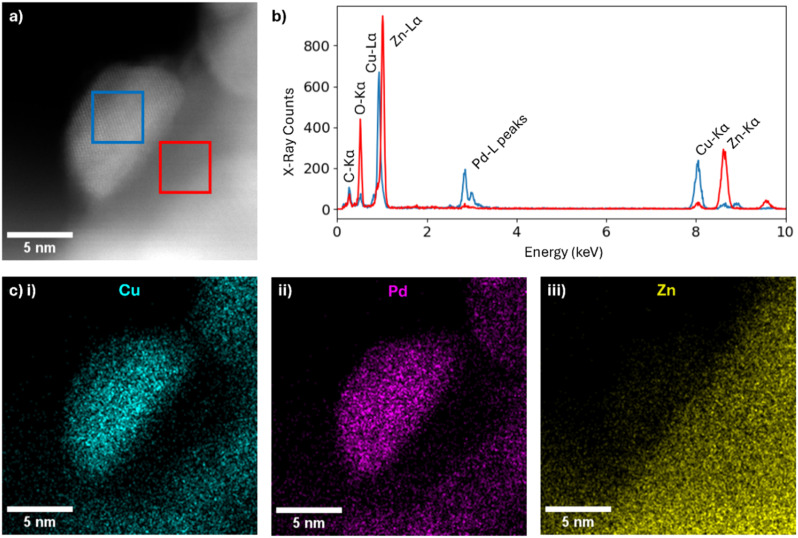
Representative HAADF-STEM imaging and EDX characterisation. (a) HAADF-STEM image of one nanoparticle in Pd_0.50_Cu_0.50_ with two areas indicated with red and blue squares; (b) EDX spectra (red and blue) for the areas indicated by the red and blue squares respectively in (a); (c) EDX elemental maps of the nanoparticle shown in (a), mapping (i) Cu-K_α_, (ii) Pd-L_α_ and (iii) Zn-K_α_ peaks.

Previous studies have shown that PdZn can suppress CH_4_ formation, thereby enhancing methanol selectivity.^38^ To further determine whether methane suppression in our system arises from PdCu or PdZn alloy formation, we performed scanning transmission electron microscopy (STEM) combined with energy dispersive X-ray (EDX) spectroscopy for c-Pd_0.50_Cu_0.50_ (Fig. 4).

Fig. 4c shows a homogeneous distribution of Pd and Cu within the bimetallic particles, indicating a high degree of alloying, consistent with XPS and DRIFTS results (Fig. 2) and the bimetallic assembly mechanism proposed (Fig. 1a). Importantly, Zn was confined to the support, with no evidence of incorporation into the catalyst particles except in the interface between Pd and ZnO support as confirmed by our DFT calculations as well (Fig. 4b, c and S1). EDX spectra of the catalyst region (blue area) and the underlying support (red area) confirmed the absence of intermixing between the Zn and the PdCu bimetallic particles.

The presence of Cu appears to effectively inhibit the inclusion of Zn in the PdCu structure, which has previously been identified as the main factor responsible for CH_4_ suppression in monometallic Pd systems. These results demonstrate that with an appropriate Pd-to-Cu ratio, PdCu bimetallics can also suppress methane formation. This is evident in c-Pd_0.50_Cu_0.50_, c-Pd_0.25_Cu_0.75_, and c-Pd_0.12_Cu_0.88_, all of which show negligible CH_4_ production. Critically, for all PdCu catalysts, no loss of activity or signs of deactivation were observed over the testing period (*ca.* 100 hours), highlighting the stability of the catalysts. Furthermore, c-Pd_0.25_Cu_0.75_ and c-Pd_0.75_Cu_0.25_ were tested for three catalytic cycles (Fig. S19), with no loss of methanol productivity observed upon retesting. Among all catalysts tested, c-Pd_0.50_Cu_0.50_ exhibited the highest methanol productivity, likely due to the synergistic effect between the intrinsically high hydrogenation activity of Pd and the high methanol selectivity characteristic of Cu-based catalysts.

The role of Cu in the PdCu system can be rationalised considering previous findings for the Pd/ZnO system, where the formation of the PdZn alloy enhanced methanol production by facilitating the formation of formate intermediates and their subsequent hydrogenation to methanol.^38,62–64^ A similar mechanistic pathway appears to operate for the PdCu alloys reported here, where the exclusion of Zn from the alloy is clearly observed (Fig. 4). Thus, the modulation of Pd through alloying with either Zn or Cu significantly influences the methanol formation rate. *In situ* DRIFTs experiment further support this mechanistic interpretation (Fig. 2f), showing that changes in the PdCu alloy composition, achieved by varying the deposition order to expose either Pd or Cu at the surface, affect methanol selectivity. Moreover, previous DFT studies have shown that PdCu alloys facilitate the facile formation of formate intermediates, further promoting methanol production during CO_2_ hydrogenation.^28^

Table S3 shows values of methanol productivity for previously reported PdCu catalysts in the literature, along with the c-Pd_0.50_Cu_0.50_ prepared in this work. It can be seen that the methanol productivity of 8.2 mol_MeOH_ h^−1^ mol_metal_ achieved by the c-PdCu catalyst assembled directly from metal atoms is considerably higher than previously reported values, despite the majority of previous studies being conducted at higher pressures, which allows for higher equilibrium yields of methanol. This demonstrates that assembling particles directly on the surface in a solvent- and reagent-free environment is not only an effective technique for improving atom economy during catalyst production (Table S4 shows that this approach significantly enhances metal utilisation efficiency) but also leads to superior productivity in the synthesis of methanol from CO_2_. This further highlights the magnetron sputtering technique as a sustainable method for the efficient production of complex bimetallic particles systems, where strict control over composition and atomic mixing plays a significant role in determining catalyst activity.

## Conclusions

This study has demonstrated that directly depositing metals in atomic form onto a support is a highly efficient, versatile and sustainable method for fabricating bimetallic catalysts. This approach enabled the formation of PdCu/ZnO catalysts that exhibit increased activity for CO_2_ hydrogenation. Importantly, it was shown that different metals can be effectively mixed at the atomic scale in desired ratios to produce bimetallic PdCu/ZnO catalysts. Interestingly, the order in which metal atoms are added significantly impacted catalytic activity. When Pd and Cu were co-deposited onto ZnO, the resulting catalyst exhibited the highest productivity for methanol as compared to catalysts produced by sequential deposition and to any previously reported PdCu catalysts. This demonstrates the considerable tunability of catalyst composition and corresponding performance, which can be achieved simply by varying how metal atoms are mixed on the surface. The presence of Cu enhances CO_2_ adsorption, inhibits the inclusion of Zn in PdCu particles and modulates the binding strength of Pd to reaction intermediates leading to negligible CH_4_ formation and, resulting in high selectivity for methanol production while maintaining the high CO_2_ conversion characteristic of Pd. Under the given reaction conditions, we observed no loss of performance or signs of catalyst deactivation. The catalyst preparation method using magnetron sputtering demonstrates, for the first time, that not only the Pd : Cu ratio but also the sequence of metal deposition plays a critical role in producing a superior bimetallic catalyst for the thermal conversion of CO_2_ to methanol.

## Methods

### Catalyst preparation

All depositions were conducted using a custom-built AJA magnetron sputtering system. Samples were mounted on a specially designed rotating holder to ensure continuous agitation during the deposition process. This assembly was loaded into the pre-chamber of the sputtering system, which reached a base pressure of 3 × 10^−7^ torr within 30 minutes. The sample holder was then transferred to the main chamber, where a background pressure of 3 × 10^−8^ torr was maintained. Upon closing the gate valve to isolate the main chamber from the pre-chamber, the system required approximately 5 minutes to re-establish the base pressure of 3 × 10^−8^ torr. Metal deposition was performed at room temperature using high-purity argon (10 mTorr) as the working gas. Depending on the experimental design, either Pd (99.99%) or Cu (99.99%), or both, were used in monometallic or bimetallic configurations. Two deposition modes were employed: (1) co-deposition, in which both metals were deposited simultaneously onto the support. Samples prepared by this method were labelled with the prefix “c-”, *e.g.*, c-PdCu; and (2) sequential deposition, where one metal was deposited prior to the other. These samples were labelled with the prefix “s-”, and the order of the elements in the label reflects the deposition sequence. For example, in s-CuPd, Cu was deposited before Pd. Additionally, the Cu/Pd atomic ratio was calibrated in advance by adjusting the sputtering power applied to each target. The metal loading of the catalysts was quantified using inductively coupled plasma–optical emission spectroscopy (ICP-OES) on a PerkinElmer Optima 2000 spectrometer. For sample preparation, approximately 10 mg of catalyst powder was subjected to microwave-assisted acid digestion in 2 mL of freshly prepared aqua regia (a 3 : 1 mixture of hydrochloric and nitric acids). The digestion was carried out at 150 °C for 1 hour to ensure complete dissolution of both metals and zinc oxide components. After cooling, the digested samples were diluted to a final volume of 10 mL using a 5% (v/v) hydrochloric acid solution to stabilise the metal ions before analysis.

### DFT calculations

Spin-polarised Density Functional Theory (DFT) calculations of binding energies of metal atoms to zinc oxide were performed with the Vienna *Ab initio* Simulation Package (VASP), within the plane-wave projector augmented-wave (PAW) method. The structures were relaxed using the Perdew–Burke–Ernzerhof (PBE) exchange–correlation functional with a force tolerance of 0.01 eV A^−1^ and an electronic convergence criteria of 10^−6^ eV. The energy cut-off was set to 500 eV, and a Monkhorst–Pack *k*-point grid of 4 × 4 × 1 was used to sample the Brillouin zone. van der Waals interactions were taken into account using the DFT-D3 method with Becke–Jonson damping function.

### DRIFTS under reaction conditions

CO_2_ hydrogenation to methanol reaction was investigated by *operando* Diffuse Reflectance Infrared Fourire Transform Spectroscopy (DRIFTS). Measurements were performed with a IS50 ThermoFisher Scientific Spectrometer equipped with a MCT detector, a KBr beam splitter, and a flow-through high temperature environmental cell—the TPR Praying Mantis™ Diffuse Reflection Accessory (HR-DRP-BR3) with ZnSe windows—capable of operating at high temperatures and pressure (up to 800 °C and 34 bar). Spectra were acquired by collecting 256 scans at a resolution of 4 cm^−1^ over a scan range from 4000 to 650 cm^−1^. The cell sample holder (i.d.: 5 mm; height: 4 mm) was located within a chamber with ZnSe windows.

Prior to each catalytic tests, the sample was reduced *in situ* at 230 °C and atmospheric pressure for 1 h in flux (20 mL min^−1^) of 5% H_2_/Ar (heating rate *β* = 5 °C min^−1^). Then the catalyst was purged with Ar at 270 °C and a background spectrum was acquired. The temperature was reduced to 125 °C and the reaction mixture of CO_2_/H_2_/N_2_ (23/69/8 mol mol^−1^) was introduced to the cell and the pressure increased to 20 bar. Then the reaction temperature was increased to 270 °C and the evolution of intermediates was monitored until the stabilization of the IR signals (1 h).

### Catalyst testing

The catalyst testing was undertaken using a fixed bed, continuous flow, 16-bed high throughput catalytic reactor which was designed, manufactured and serviced by Integrated Lab Solutions GmbH (ILS). The reactor was automated using Integrated Workflow Manager software, based on the LabVIEW package, and operated by the Siemens Win CC program. The reactor was divided into 4 heating blocks each containing 4 catalyst beds. In every reaction, one bed in each block was kept as a blank to ensure comparability. A capillary distribution system coupled with Equilibar back pressure regulators were used to control the gas feed and reactor pressure. A thermocouple was installed in each heating block to control the temperature.

The catalyst pellets (0.5 g unless otherwise specified, 425–600 μm pellet size) were mixed with silicon carbide (F80, 190 μm mean particle size) and centred in the isothermal zone of the stainless steel reactor tubes, which had an internal diameter of 4.0 mm. A bed of silicon carbide (F24, 750 μm mean particle size) was used at each end to limit mass transfer, and the reactor tube was plugged with quartz wool. Prior to testing, the catalysts were reduced *in situ* at 230 °C or 400 °C for 1 hour, with a ramp rate of 5 °C min^−1^, under a flow of 5% H_2_/N_2_ (40 mL min^−1^ flow rate). The reactor temperature was then cooled to 125 °C and the catalysts were held under N_2_. The system was then pressurised to 20 bar and switched to the reactant gas feed of 20% CO_2_, 60% H_2_, 5% Ar, 15% N_2_ (30 mL min^−1^ flow rate). After a stabilisation period of 4 hours, the CO_2_ hydrogenation reaction was conducted using the previously reported temperatures of 230–270 °C. A downstream purge of N_2_ (30 mL min^−1^) was used to prevent product build up and the downstream oven was held at 120 °C to avoid the condensation of the products.

The reaction products were analysed by online gas chromatography, utilising an Agilent 7890B system with two flame ionisation detectors (FIDs) and one thermal conductivity detector (TCD). The internal standard used was argon. The GC analysed 4 injections per temperature point per bed *via* a Vici stream selection valve. CO_2_ conversion was calculated by comparing the moles of CO_2_ in each bed to the moles of CO_2_ in the calibration run at 125 °C. The carbon balance was calculated as the sum of the carbon containing products (methanol and CO were the only products produced, with small amounts of CH_4_ over certain catalysts) and reactants in the feed divided by the sum of carbon containing reactants in the calibration runs.

## Author contributions

L. R. S.: conceptualization, data curation, investigation, methodology, writing – original draft preparation, writing – review & editing; E. C. K.: data curation, investigation, methodology, writing – review & editing; K. J. A.: data curation, formal analysis, investigation, writing – review & editing; M. S.: data curation, investigation, methodology; S. G.: data curation, investigation, methodology; A. W.: data curation, investigation, methodology; L. T. N.: data curation, investigation, methodology; I. E. G.: data curation, investigation; Y. C.: data curation, formal analysis; G. B.: data curation, investigation, methodology; C. C.: data curation, investigation, methodology; E. B.: data curation, investigation, methodology; D. J. M.: data curation, formal analysis, investigation; T. J. A. S.: data curation, investigation; A. N. K.: funding acquisition, supervision, writing – original draft preparation, writing – review & editing; J. A. F.: conceptualization, funding acquisition, methodology, supervision, writing – review & editing; G. J. H.: conceptualization, funding acquisition, methodology, supervision, writing – original draft preparation, writing – review & editing.

## Conflicts of interest

There are no conflicts to declare.

## Supplementary Material

SC-016-D5SC06681F-s001

## Data Availability

All data is available in the manuscript and the supplementary information (SI). Supplementary information is available. See DOI: https://doi.org/10.1039/d5sc06681f.
