# Supplementary material for: Atom-by-atom assembly reveals structure–performance control in PdCu catalysts for CO2 hydrogenation to methanol
Source: Chem Sci. 2025 Oct 20;16(47):22554–64. doi: 10.1039/d5sc06681f (PMC12557408; doi:10.1039/d5sc06681f)
Supplement: SC-016-D5SC06681F-s001 [file SC-016-D5SC06681F-s001.zip › pdcu_chem-sci_revised-es_clean.docx]

Atom-by-atom assembly reveals structure–performance control in PdCu catalysts for CO_2_ hydrogenation to methanol

Louise R. Smith,^[a]^ Emerson C. Kohlrausch,^[b]^ Kieran J. Aggett,^[a]^ Mario Samperi,^[c]^ Sadegh Ghaderzadeh,^[b]^ Andreas Weilhard,^[b^ Luke T. Norman,^[b]^ Isla E. Gow,^[a]^ Yifan Chen,^[b]^ Giuseppe Bonura,^[c]^ Catia Cannilla,^[c]^ Elena Besley,^[b]^ David J. Morgan,^[a]^ Thomas J. A. Slater,^[a]^ Andrei N. Khlobystov, ^[b]^ Jesum Alves Fernandes,^[b]^ Graham J. Hutchings.^[a]^*

*[a] Max Planck-Cardiff Centre on the Fundamentals of Heterogeneous Catalysis FUNCAT, Translational Research Hub, Cardiff University, Maindy Road, Cardiff CF24 4HQ, UK*

[b] School of Chemistry, University of Nottingham, University Park, Nottingham NG7 2RD, UK

*[c]* *CNR-ITAE Istituto di Tecnologie Avanzate per l’Energia Nicola Giordano, Via Comunale S. Lucia 5, 98126, Messina, Italy*

*Corresponding author. E-mail: [hutch@cardiff.ac.uk](mailto:hutch@cardiff.ac.uk)

**Supplementary Methods**

***Materials***

Zinc oxide (ZnO, >99.99 %) powder and palladium(II) acetylacetonate (Pd(acac)_2_, > 99.9 %) were purchased from Merck and used without further purification. The palladium (Pd, 99.99 %) and copper (Cu, 99.99 %) targets used for magnetron sputtering were purchased from Kurt J. Lesker. Hydrogen (H_2_, 99.999 %, nitrogen (N_2_, 99.998 %), carbon dioxide (CO_2_, 99.995 %), and argon (Ar, 99.998 %) were all purchased from BOC.

***Catalyst characterisation***

*XPS*

X-Ray photoelectron spectroscopy (XPS) measurements were performed using a Kratos Axis Ultra DLD system using monochromatic Al Ka X-ray source (photon energy = 1486.6 eV) operating at 144 W (12 mA x 12 kV). Data was collected with pass energies of 160 eV for survey spectra, and 40 eV for the high-resolution scans with step sizes of 1 eV and 0.1 eV respectively. The system was operated in the Hybrid mode, using a combination of magnetic immersion and electrostatic lenses, and acquired over an area approximately 300 ´ 700 µm2. A magnetically confined charge compensation system using low energy electrons was used to minimize charging of the sample surface, and all spectra were taken with a 90° take of angle. A pressure of ca. 5´10-9 Torr was maintained during collection of the spectra.

For analysis, all samples were pressed on to double sided adhesive tape attached to a glass microscope slide, itself attached to a Kratos standard sample bar.

All data was analysed using CasaXPS (v2.3.26) after subtraction of a Shirley background and using modified Wagner sensitivity factors as supplied by the instrument manufacturer. Where required, curve fits were performed using a Voigt type function (LA line shape in CasaXPS) and utilising lines shapes taken from bulk materials. Peaks positions were calibrated to the C(1s) peak of adventitious carbon,^1^ with a secondary reference to the Zn 2p_3/2_ spectra. Experimental error for peak positions was 0.2 eV.

*TEM*

Bright filed transmission electron microscopy (BF-TEM) images were acquired at 200 kV accelerating voltage on JEOL 2100F FEG TEM with a Gatan Model 1027 K3-IS direct detection camera (point resolution limit 0.23 nm, lattice resolution limit 0.1 nm).

*AC-STEM*

Aberration-corrected scanning transmission electron microscopy (AC-STEM) was conducted at the Cardiff University Electron Microscopy Facility. A probe corrected Thermo-Scientific Spectra 200 microscope was used at an operating voltage of 200 kV and a convergence semi-angle of 30 mrad. The HAADF detector had an inner collection angle of 56 mrad and an outer angle of 200 mrad. The EDX spectra were collected using four Super-X detectors.

*CO-DRIFTS*

CO-DRIFTS experiments were performed on a Bruker Tensor 27 FTIR spectrometer equipped with a MCT detector cooled using liquid nitrogen. Spectra were recorded with 64 scans per spectrum and a 4 cm^−1^ resolution. Samples were pretreated with N_2_ for 60 minutes to remove surface adsorbates before being saturated with CO (2% CO/He) for a period of 20 minutes while spectra were recorded. Following this, the CO flow was stopped and switched to N_2_, allowing for the removal of gas-phase CO, and spectra were collected over a period of 40 minutes.

*CO_2_ TPD*

100 mg of the catalyst sample was loaded into a quartz reactor. The sample was first heated to 300°C at rate of 10 K min^-1^ under a flow of 30 mL min^-1^ 5% H_2_ in Ar. After reduction for 1 h, the gas flow was switched to 30 mL min^-1^ or 1 h to purge residual hydrogen, and the sample was then cooled to 70°C. Subsequently, the sample was exposed to 30 mL min^-1^ 5% CO_2_ in Ar for 2 h to allow CO_2_ adsorption. The feed was then switched back to He for 30 min to remove physiosorbed CO_2_. Finally, the temperature was increased to 850°C at a ramp rate of 8 K min^-1^ and CO and CO_2_ were monitored using a quadrupole mass spectrometer.


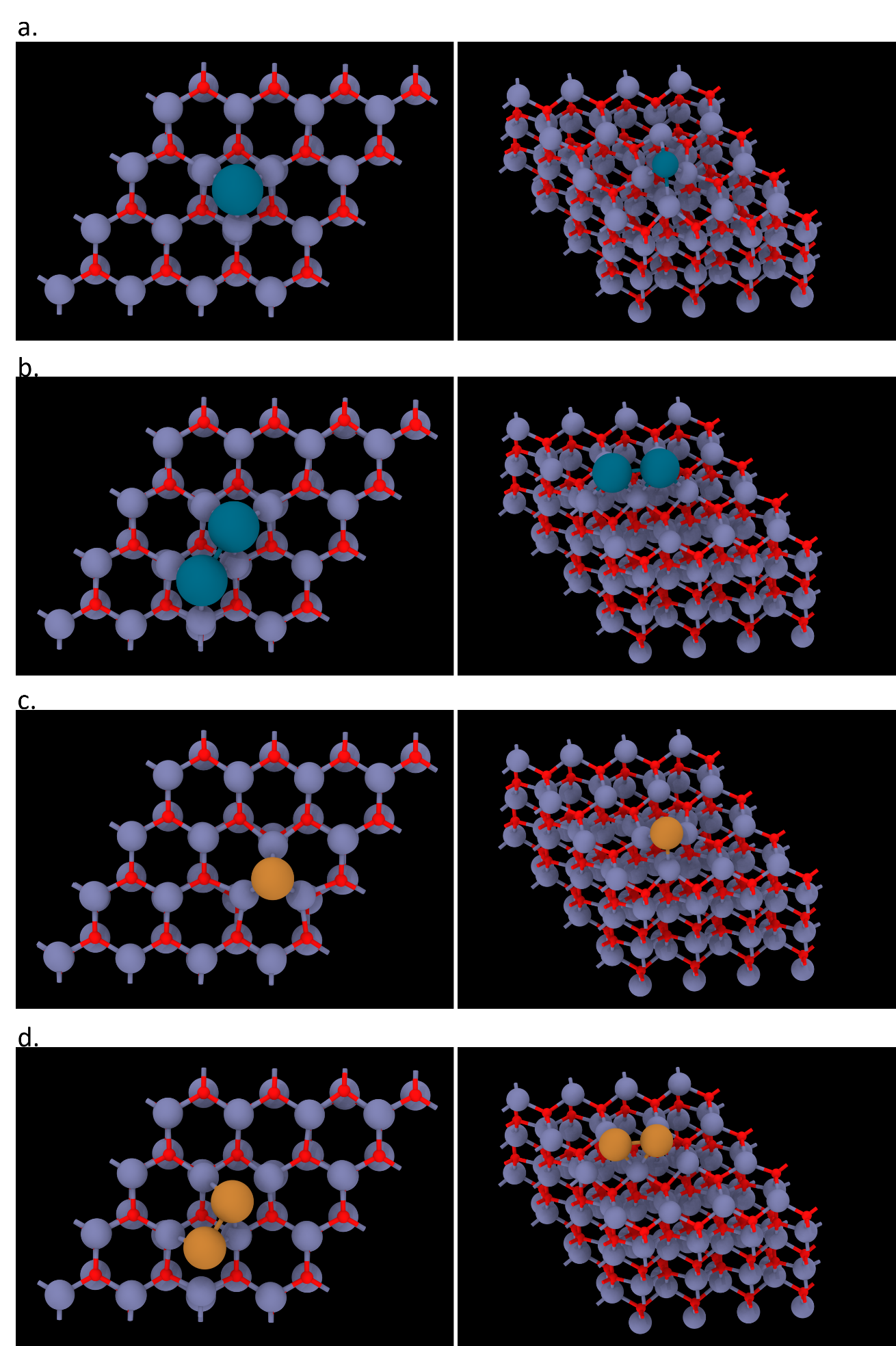


Figure S1. Density functional theory (DFT) calculations of Pd **a.** atom and **b.** dimer binding energies on ZnO (0001) facet of -4.25 and -4.13, respectively, and Cu **c.** atom and **d.** dimer binding energy on ZnO (0001) facet of -2.95 and -3.31 eV, respectively.


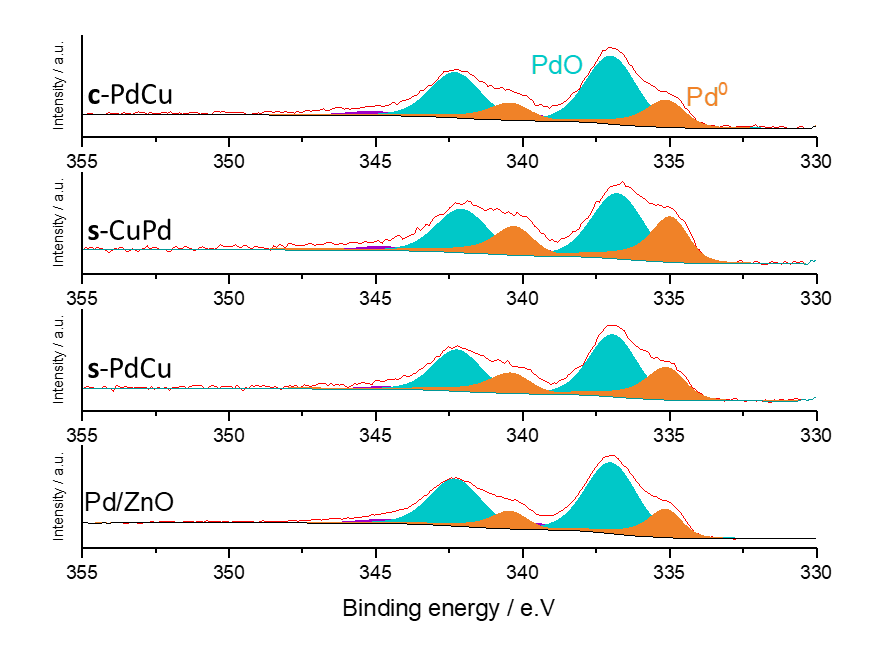


Figure S2. Pd 3d XPS spectra for as-prepared PdCu/ZnO catalysts prepared by MS with different deposition modes (simultaneous and sequential metal deposition). The spectra for Pd/ZnO is also shown for reference.

**Supplementary note 1**

Prior to reduction at 230 °C, Pd spectra of PdCu catalysts prepared by simultaneous and sequential deposition all showed the presence of Pd^0^ (represented by the orange peak at 335 ev) and PdO (represented by the blue peak at 337 eV), similar to the Pd/ZnO spectrum. No significant difference in peak position was observed for the un-reduced materials.


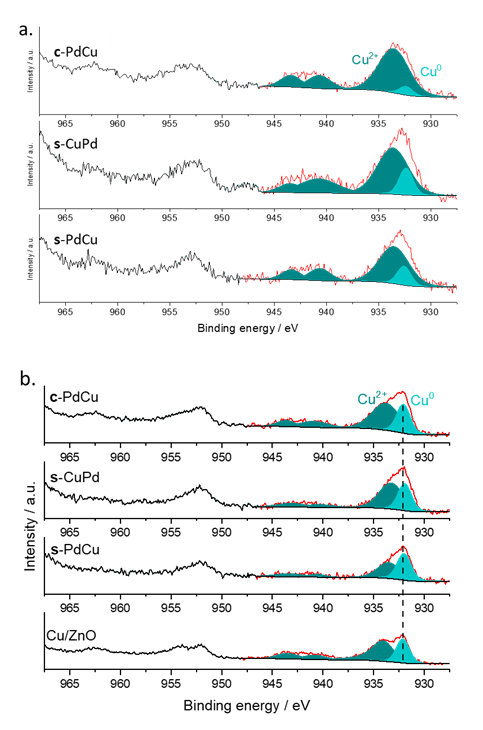


Figure S3. Cu 2p XPS spectra for PdCu/ZnO catalysts with different deposition modes (co-deposition and sequential metal deposition), **a.** as-prepared, and **b.** after reduction at 230 °C. The spectra for Cu/ZnO is also shown for reference.

Table S1. Cu 2p_3/2_ and Pd 3d_3/2_ XPS peak positions, the ratio of the Pd alloy and metallic Pd XPS peak areas, the ratio of Pd and Cu from peak areas, and the Pd&Cu surface composition from the ratios of the Pd and Cu peak areas, relative to the Zn and O peak areas.

| ***Sample (reduced)*** | ***Cu*** ***2p_3/2_ (metallic) peak position (eV)*** | ***Pd 3d_3/2_ (alloy) peak position (eV)*** | ***Pd 3d_3/2_ (metallic) peak position (eV)*** | ***Pd alloy: Pd metal ratio*** | ***Pd:Cu ratio*** | ***Pd&Cu surface composition (%)*** |
| --- | --- | --- | --- | --- | --- | --- |
| **c**-PdCu | 932.1 | 336.2 | 335.2 | 0.46 | 0.75 | 4.33 |
| **s**-PdCu | 932.0 | 336.4 | 335.2 | 0.16 | 0.93 | 2.12 |
| **s**-CuPd | 931.9 | 336.0 | 335.0 | 0.43 | 0.72 | 2.32 |
| Pd | - | 335.8 | 334.8 | 0.79 | - | - |
| ***Sample (as-prepared)*** |  | ***Pd 3d_3/2_ (PdO) peak position (eV)*** | ***Pd 3d_3/2_ (metallic) peak position (eV)*** |  |  |  |
| **c**-PdCu | 932.4 | 337.0 | 335.0 | - | 0.99 | 3.77 |
| **s**-PdCu | 932.6 | 336.9 | 335.0 | - | 0.99 | 2.25 |
| **s**-CuPd | 932.4 | 336.8 | 334.9 | - | 0.86 | 2.36 |
| Pd | - | 337.0 | 335.0 | - | - |  |


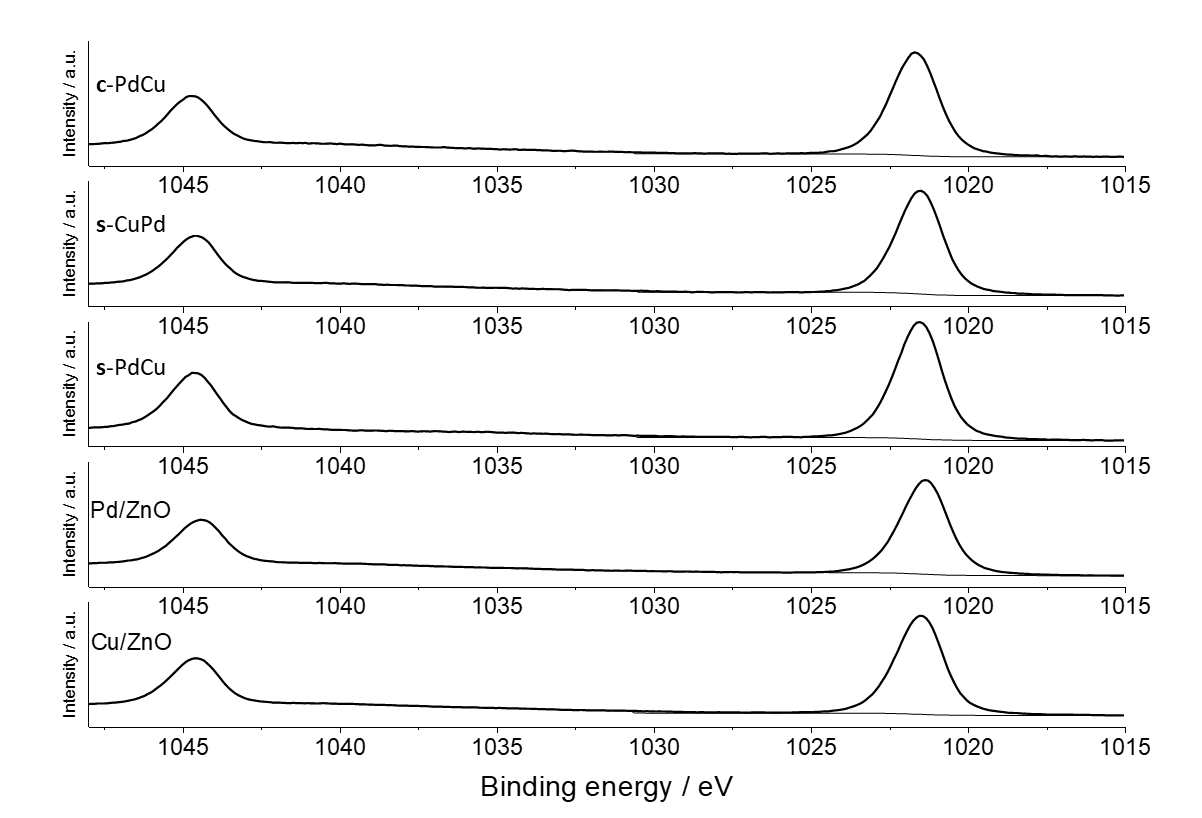


Figure S4. Zn 2p XPS spectra for PdCu/ZnO prepared by MS with different deposition modes (simultaneous and sequential metal deposition) after reduction at 230 °C. The spectra for Pd/ZnO and Cu/ZnO are also shown for reference.


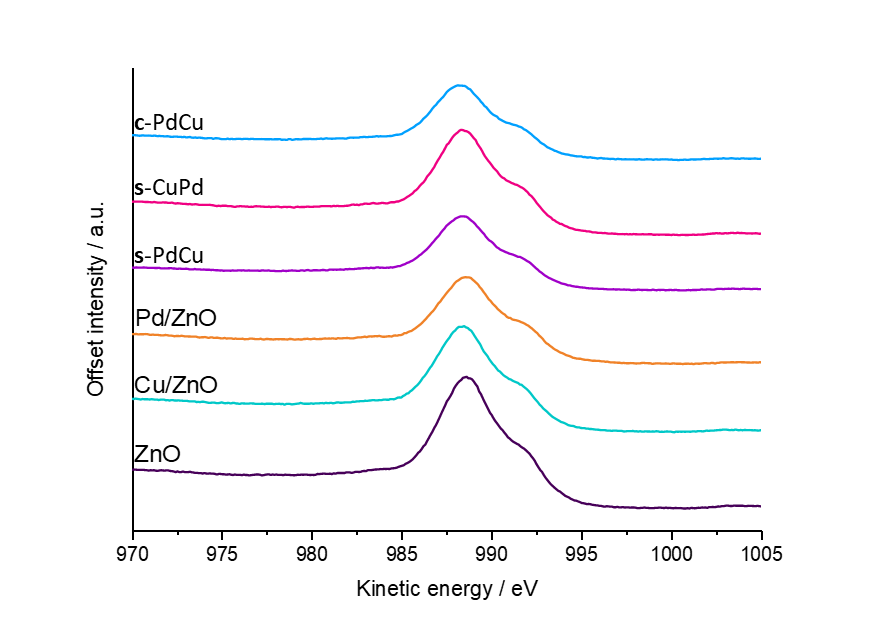


Figure S5. Zn LMM spectra for PdCu/ZnO prepared by MS with different deposition modes (simultaneous and sequential metal deposition) after reduction at 230 °C. The spectra for Pd/ZnO, Cu/ZnO and the ZnO support are also shown for reference.

**Supplementary note 2**

For Cu/ZnO, Pd/ZnO and PdCu/ZnO prepared by a variety of deposition methods, the Zn LMM spectra are dominated by ZnO (also shown for reference), at 988.5 eV with no evidence of metallic Zn present. The LMM spectra for metallic Zn shows a peak with a maximum at 992 eV, which may not be visible in the presence of ZnO due to the shoulder at the same kinetic energy (as shown above) but an additional peak at 996.0 eV can be used to distinguish between ZnO and metallic Zn. Here, no evidence of metallic Zn exists.


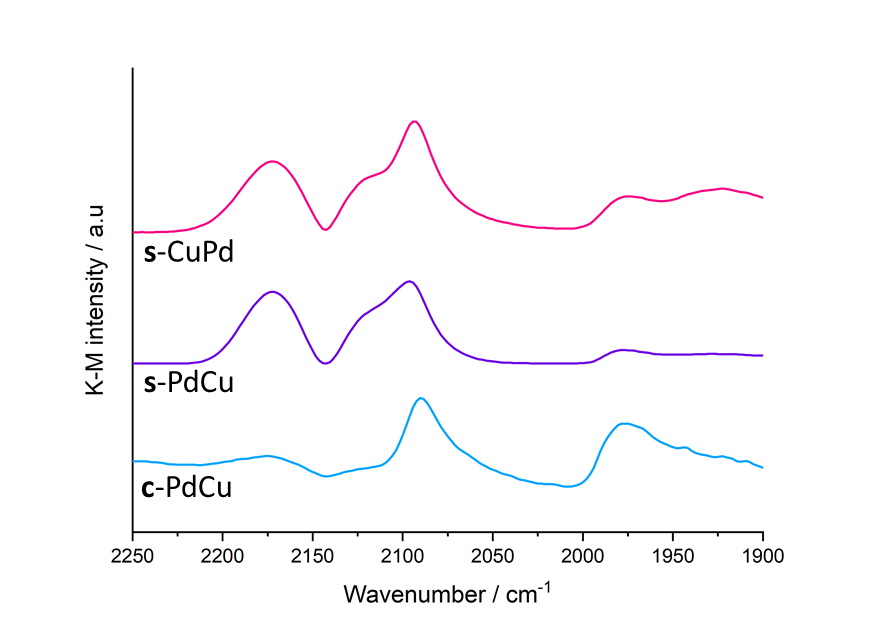


Figure S6. DRIFT spectra for PdCu/ZnO prepared by MS with different deposition modes (simultaneous and sequential metal deposition). Spectra obtained under flowing CO (2 % CO/He).

Figure S7. Temperature-programmed desorption (TPD) of CO₂ was conducted to investigate the adsorption behaviour of CO₂ on the catalyst surfaces.


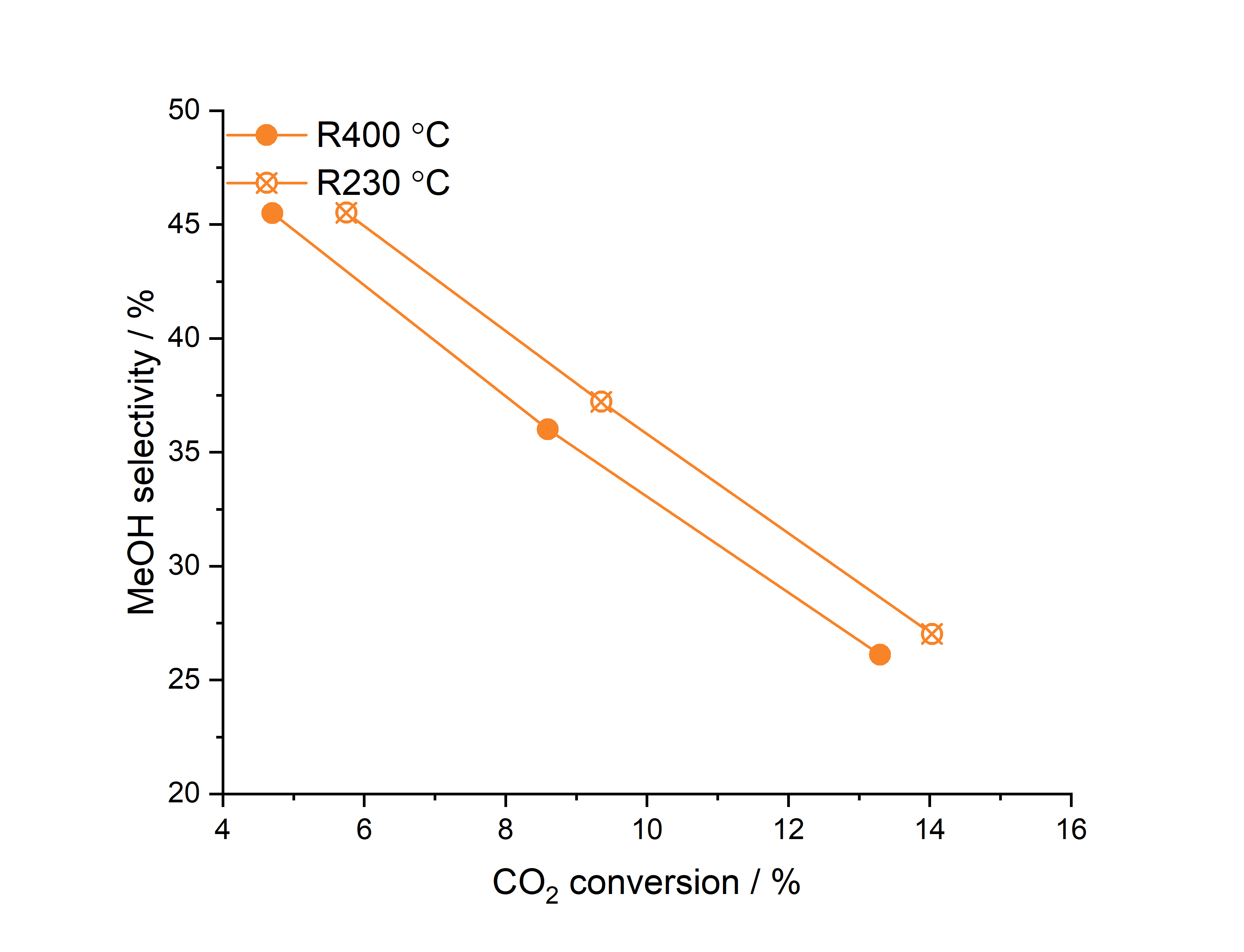


Figure S8. Methanol selectivity *vs.* CO_2_ conversion for 1 wt.% Pd/ZnO catalysts (0.5 g) prepared by MS and reduced *in situ* at 400 °C (solid symbols) or 230 °C (open cross symbols) prior to testing at 230, 250 and 270 °C with a CO_2_:H_2_ ratio of 1:3 and a total pressure of 20 bar.

**Supplementary note 3**

Prior to testing the physical mixture of Pd/ZnO and Cu/ZnO, the Pd/ZnO catalyst was reduced at 230 °C to confirm that the lower reduction temperature did not result in a detrimental effect on catalyst productivity, as all PdCu catalysts were reduced at 230 °C. A slight increase in activity was observed for the catalyst reduced at the lower temperature which was reflected in an increase in methanol productivity (figure S16).


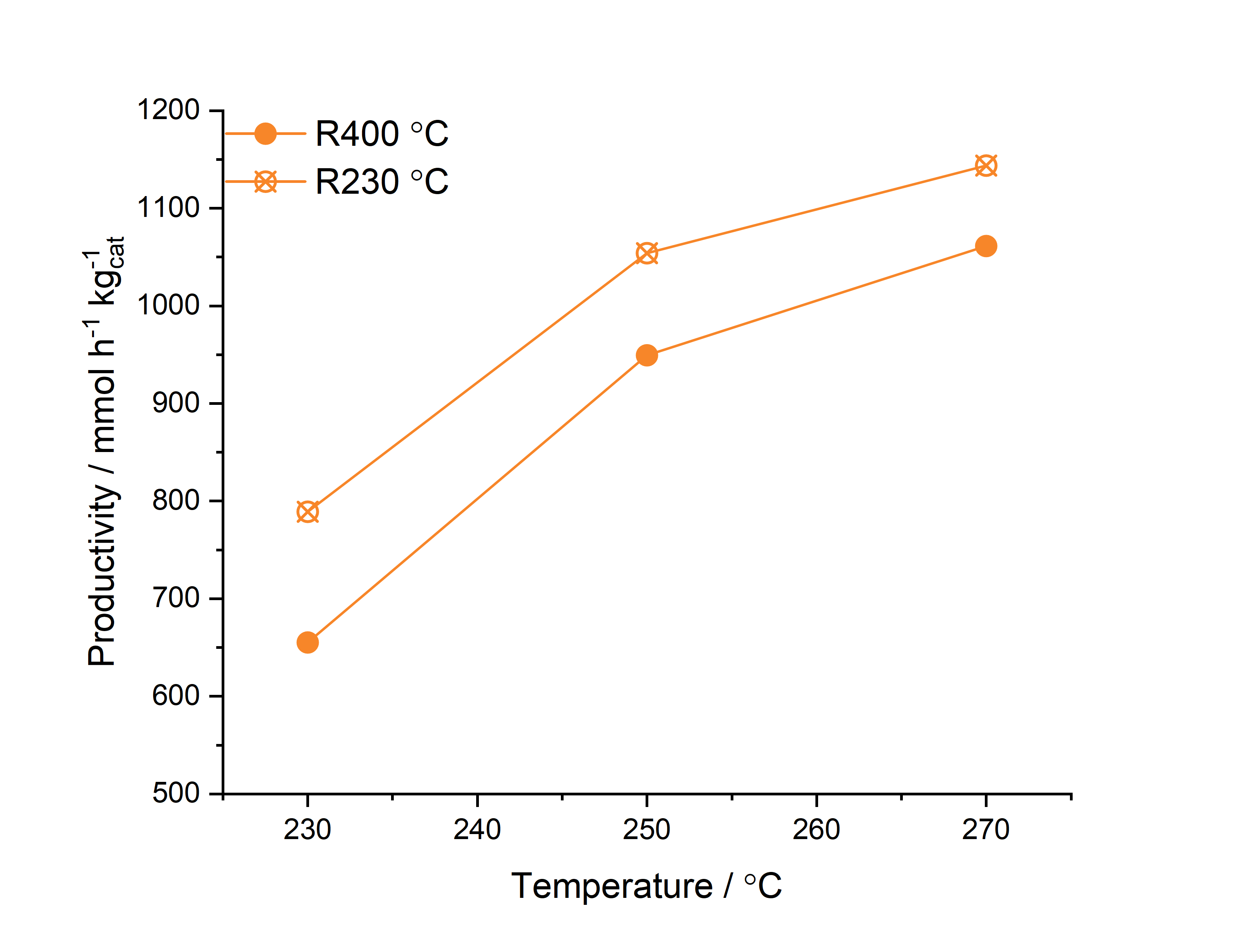


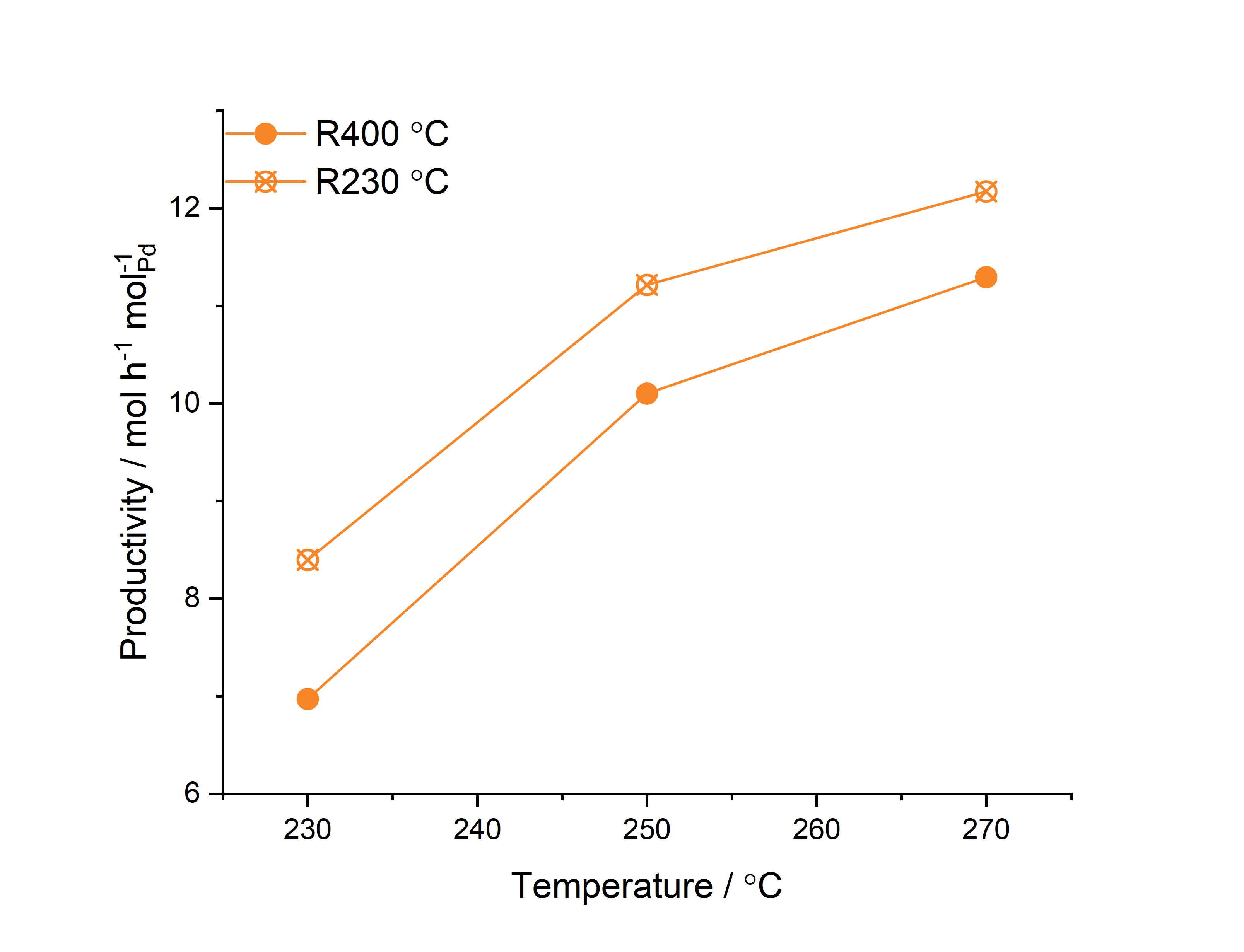


Figure S9. Methanol productivity as a function of reaction temperature for 1 wt.% Pd/ZnO catalysts (0.5 g) prepared by MS and reduced *in situ* at 400 °C (solid symbols) or 230 °C (open cross symbols) prior to testing at 230, 250 and 270 °C with a CO_2_:H_2_ ratio of 1:3 and a total pressure of 20 bar.


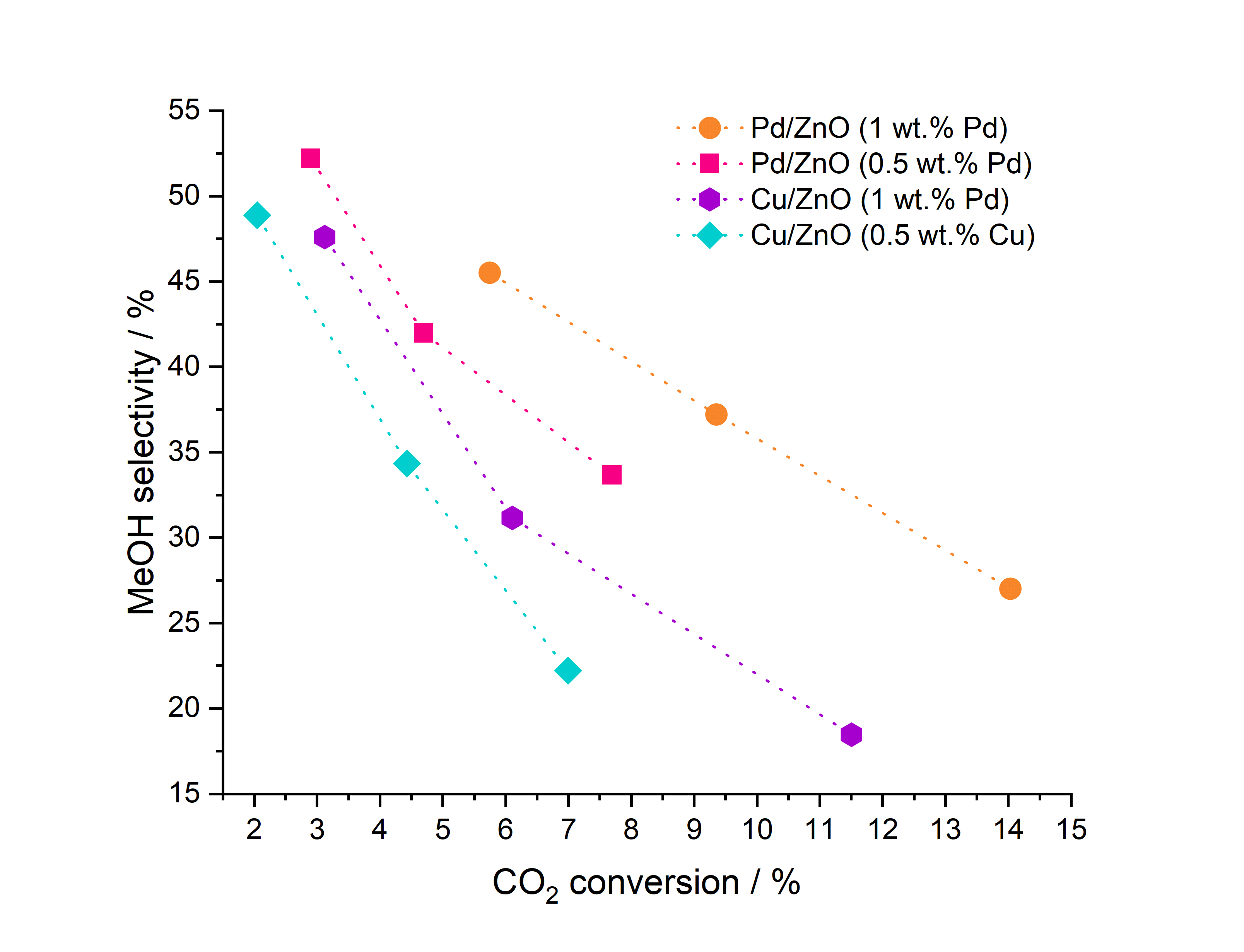


Figure S10. Methanol selectivity *vs.* CO_2_ conversion for Pd/ZnO and Cu/ZnO catalysts (0.5 g) with total loadings of 0.5 or 1 wt.% as specified in the legend, prepared by MS and reduced *in situ* at 230 °C prior to testing at 230, 250 and 270 °C with a CO_2_:H_2_ ratio of 1:3 and a total pressure of 20 bar.


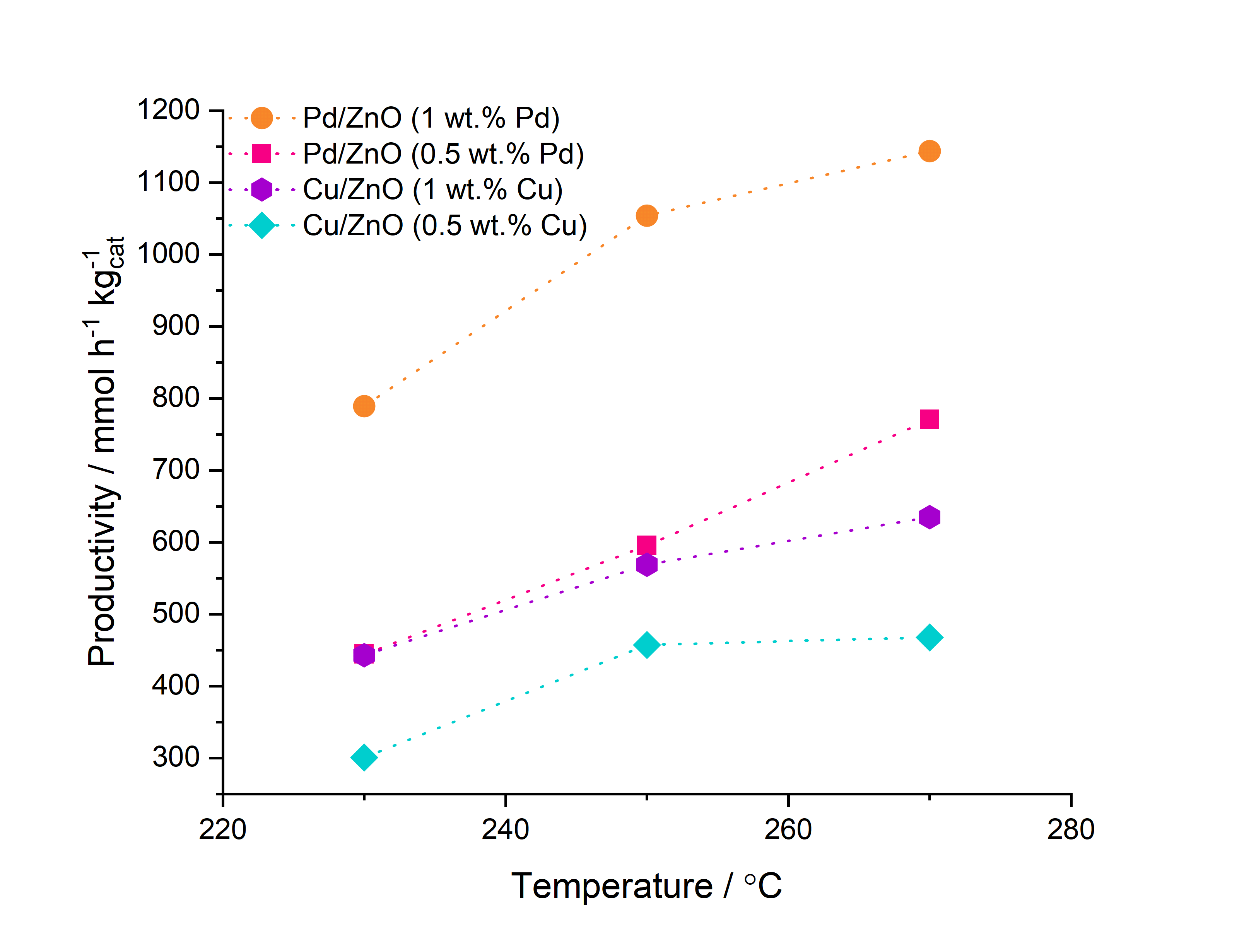


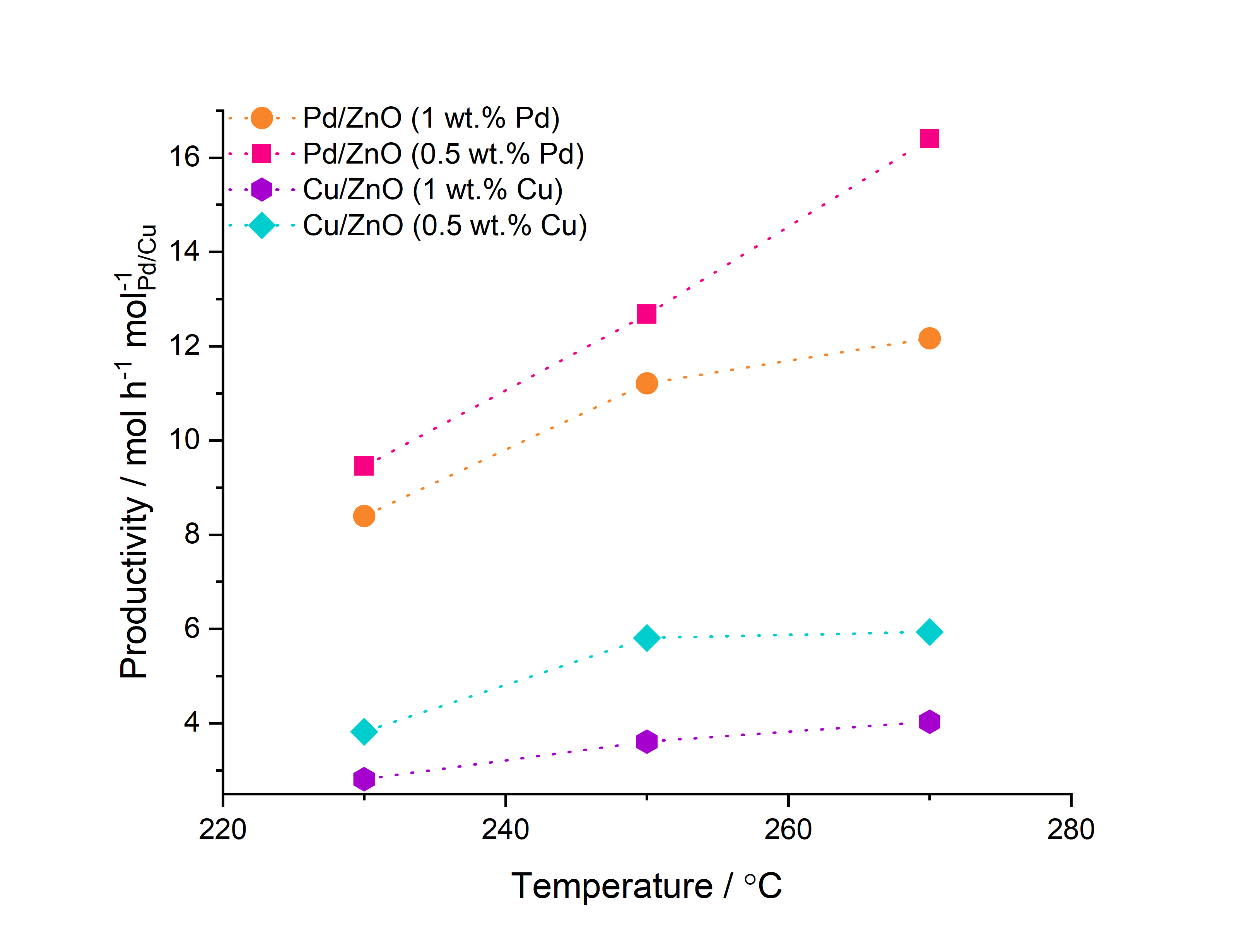


Figure S11. Methanol productivity as a function of reaction temperature for Pd/ZnO and Cu/ZnO catalysts (0.5 g) with total loadings of 0.5 or 1 wt.% as specified in the legend, prepared by MS and reduced *in situ* at 230 °C prior to testing at 230, 250 and 270 °C with a CO_2_:H_2_ ratio of 1:3 and a total pressure of 20 bar.


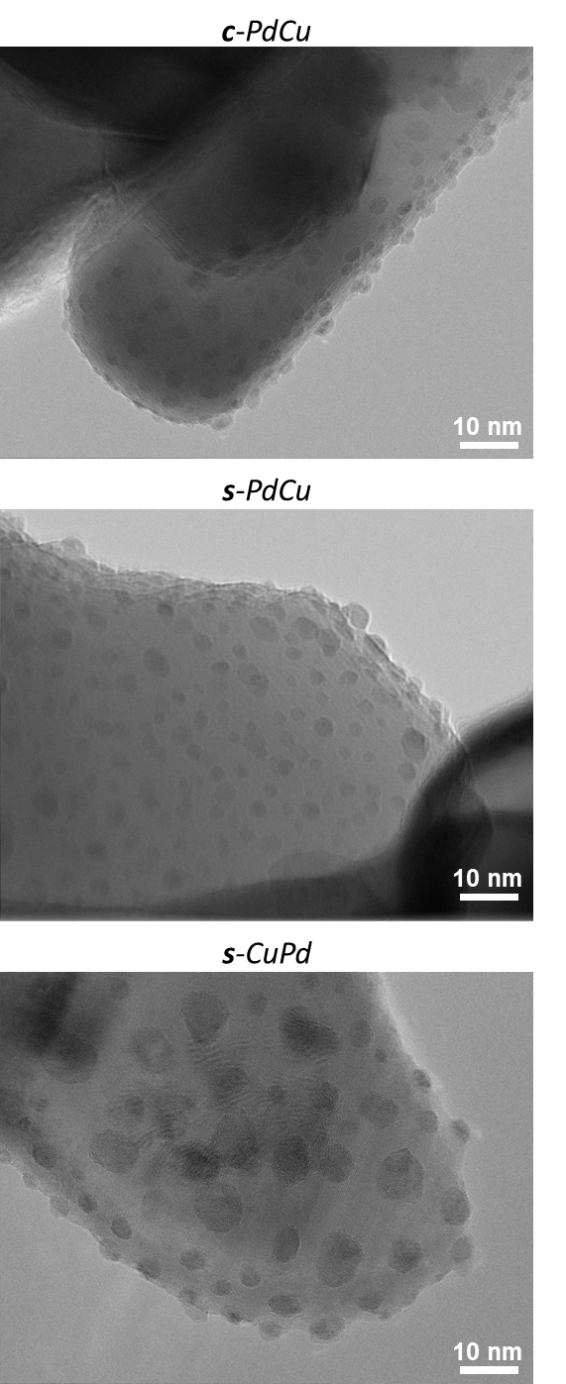


Figure S12. HR-TEM images of **c**-PdCu, **s**-PdCu, and **s**-CuPd catalysts show no significant changes in mean particle diameter or size distribution after catalysis.

Figure S13. Pre-reaction DRIFTS of the investigated catalysts. (a)-(c) DRIFT spectra of the investigated catalysts prior to reduction and (d)–(e) after reduction and Ar purge at 270°C.

Figure S14. (a) Overview of the acquired DRIFT spectra recorded under reaction conditions (20 bar, 270°C), highlighting CO_2_ vibrations at 2349 cm^-1^ and 3500 cm^-1^-3800 cm^-1^ and the region of CO vibrations.

Table S2. CH_4_ selectivity at different reaction temperatures for PdCu/ZnO catalysts with a range of PdCu compositions. The catalysts are denoted by the weight loadings of Pd and Cu. Data for monometallic Pd/ZnO and Cu/ZnO with loadings of 1 wt.% are also shown for reference.

| ***PdCu catalyst*** | ***Reaction Temperature (°C)*** | ***CH_4_ selectivity (%)*** |
| --- | --- | --- |
| Pd | 230 | <0.1 |
|  | 250 | <0.1 |
|  | 270 | <0.1 |
| Pd_0.75_Cu_0.25_ | 230 | 0.78 |
|  | 250 | 0.65 |
|  | 270 | 0.51 |
| Pd_0.50_Cu_0.50_ | 230 | <0.1 |
|  | 250 | <0.1 |
|  | 270 | <0.1 |
| Pd_0.25_Cu_0.75_ | 230 | <0.1 |
|  | 250 | <0.1 |
|  | 270 | <0.1 |
| Pd_0.12_Cu_0.88_ | 230 | <0.1 |
|  | 250 | <0.1 |
|  | 270 | <0.1 |
| Pd_0.05_Cu_0.95_ | 230 | 0.53 |
|  | 250 | 0.35 |
|  | 270 | 0.23 |
| Pd_0.01_Cu_0.99_ | 230 | 0.34 |
|  | 250 | 0.19 |
|  | 270 | 0.11 |
| Cu | 230 | 0.13 |
|  | 250 | 0.11 |
|  | 270 | <0.1 |


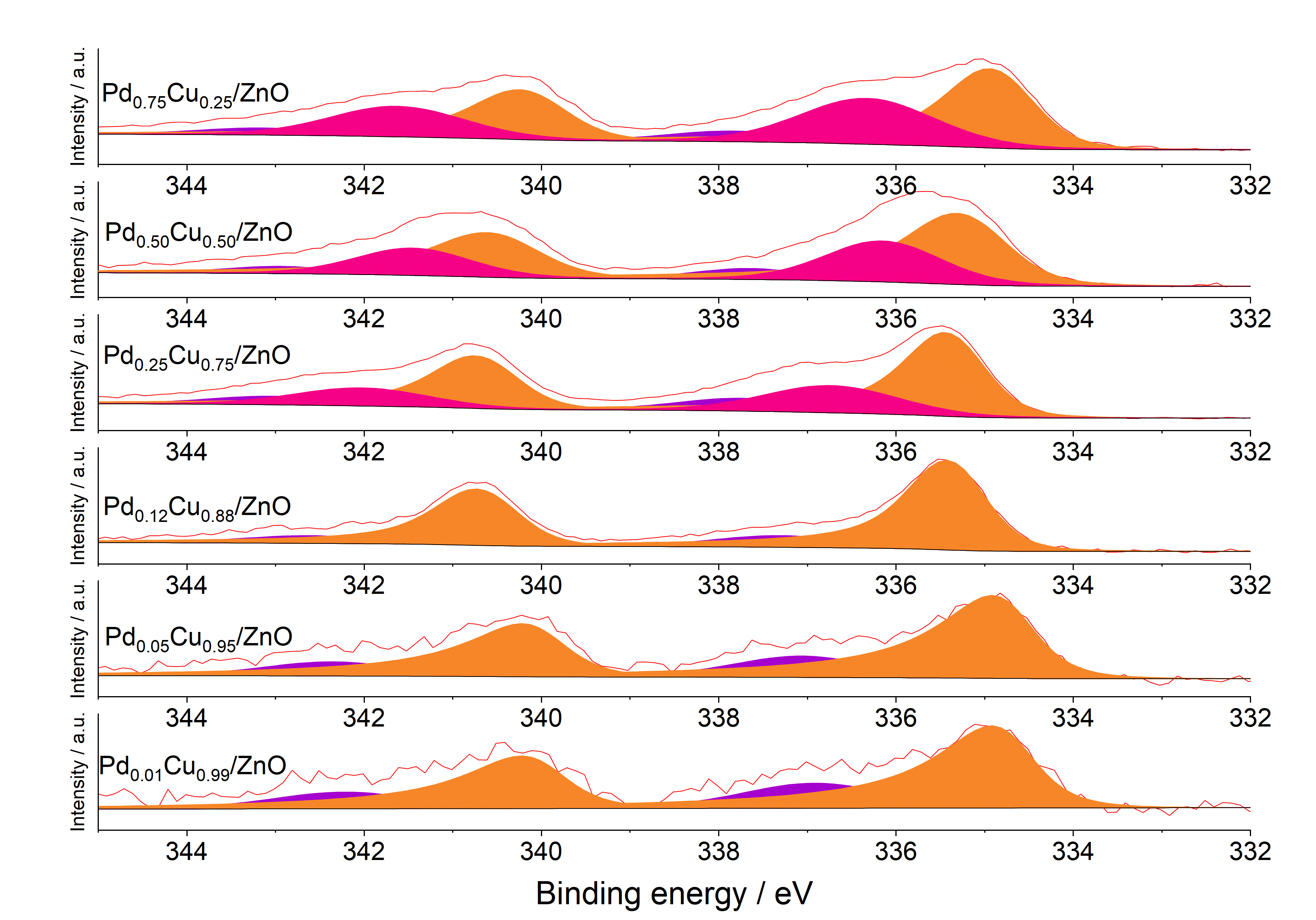


Figure S15. Pd 3d XPS spectra for PdCu/ZnO catalysts with a range of Pd:Cu ratios after reduction at 230 °C.

**Supplementary note 4**

The Pd 3d XPS spectra for PdCu/ZnO catalysts with a range of loadings show no significant changes in the binding energy of the Pd^0^ and the Pd alloy peak. The lowest binding energy of the Pd^0^ peak was observed at 334.9 eV for Pd_0.75_Cu_0.25_, Pd_0.05_Cu_0.95_, and Pd_0.01_Cu_0.99_, with a binding energy of 335.2 eV for Pd_0.50_Cu_0.50_ and 335.4 eV for Pd_0.25_Cu_0.75_ and Pd_0.12_Cu_0.88_. The peak positions were calibrated to the adventitious carbon species in the C1s spectra (figure S23) where no variation in binding energy was observed. Due to the differences in the Pd binding energies, the Zn 2p spectra (figure S22) was used as a secondary calibration. No variation outside of experimental error (0.2 eV) was observed for the Zn 2p spectra.


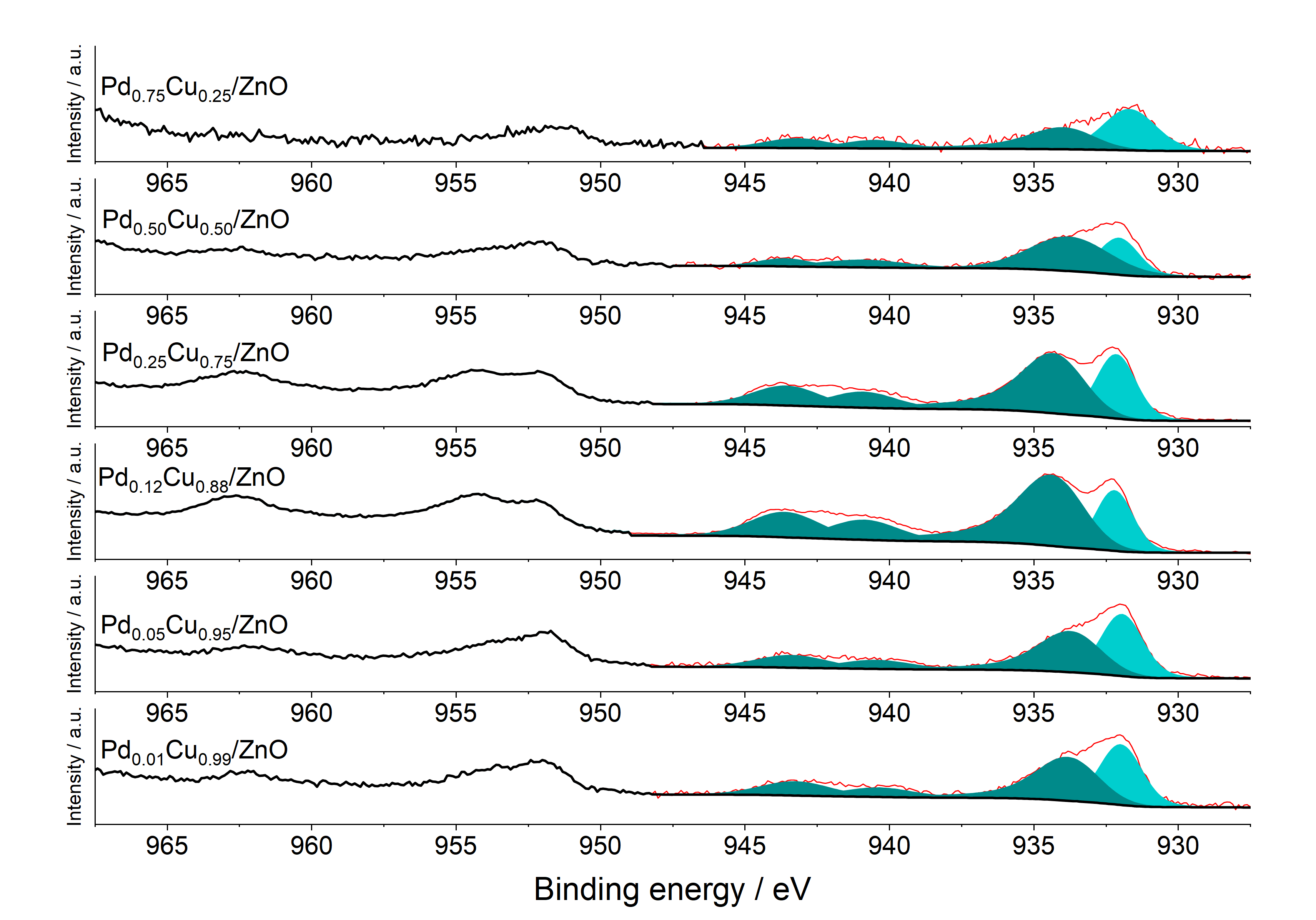
Figure S16. Cu 2p XPS spectra for PdCu/ZnO catalysts with a range of Pd:Cu ratios after reduction at 230 °C.

**Supplementary note 5**

Unlike the Pd 3d spectra, no shifts in the Cu^0^ peak were observed, except for Pd_0.75_Cu_0.25_ which was at a binding energy of 931.7 eV compared to 932.1 eV for the other samples. Some variation in the binding energy of the CuO peaks were observed between samples.


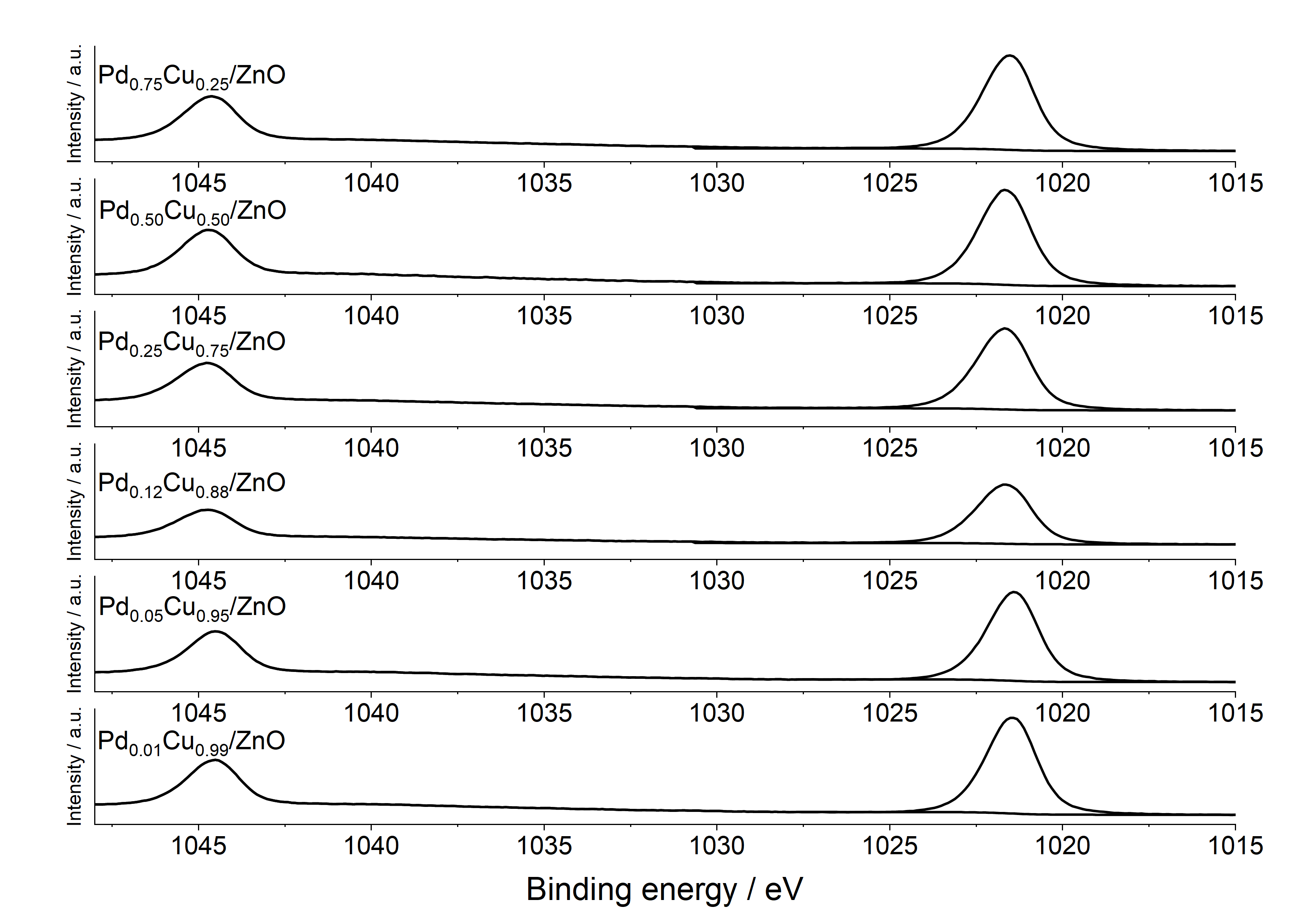


Figure S17. Zn 2p XPS spectra for PdCu/ZnO catalysts with a range of Pd:Cu ratios after reduction at 230 °C.


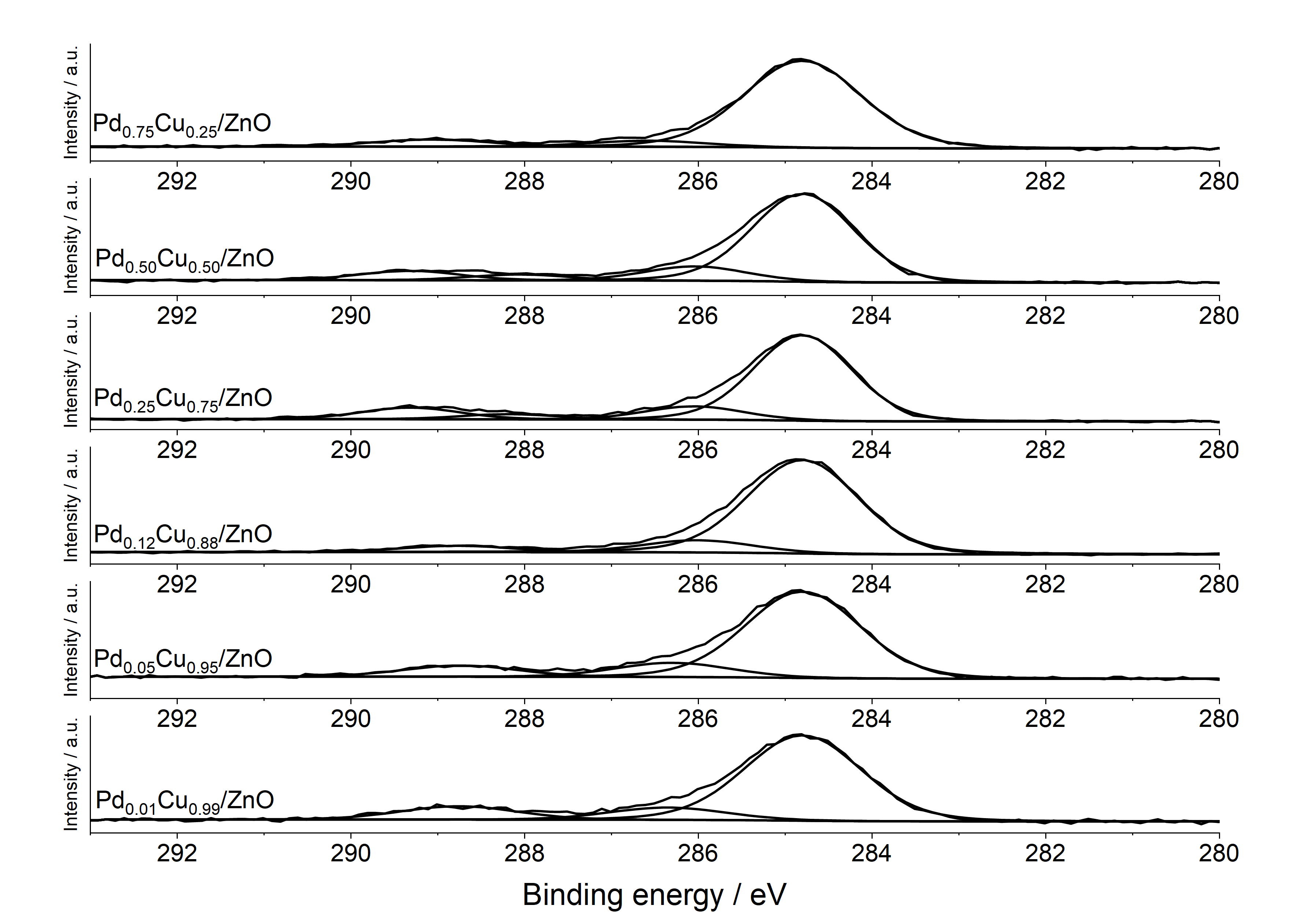


Figure S18. Cu 1s XPS spectra for PdCu/ZnO catalysts with a range of Pd:Cu ratios after reduction at 230 °C.


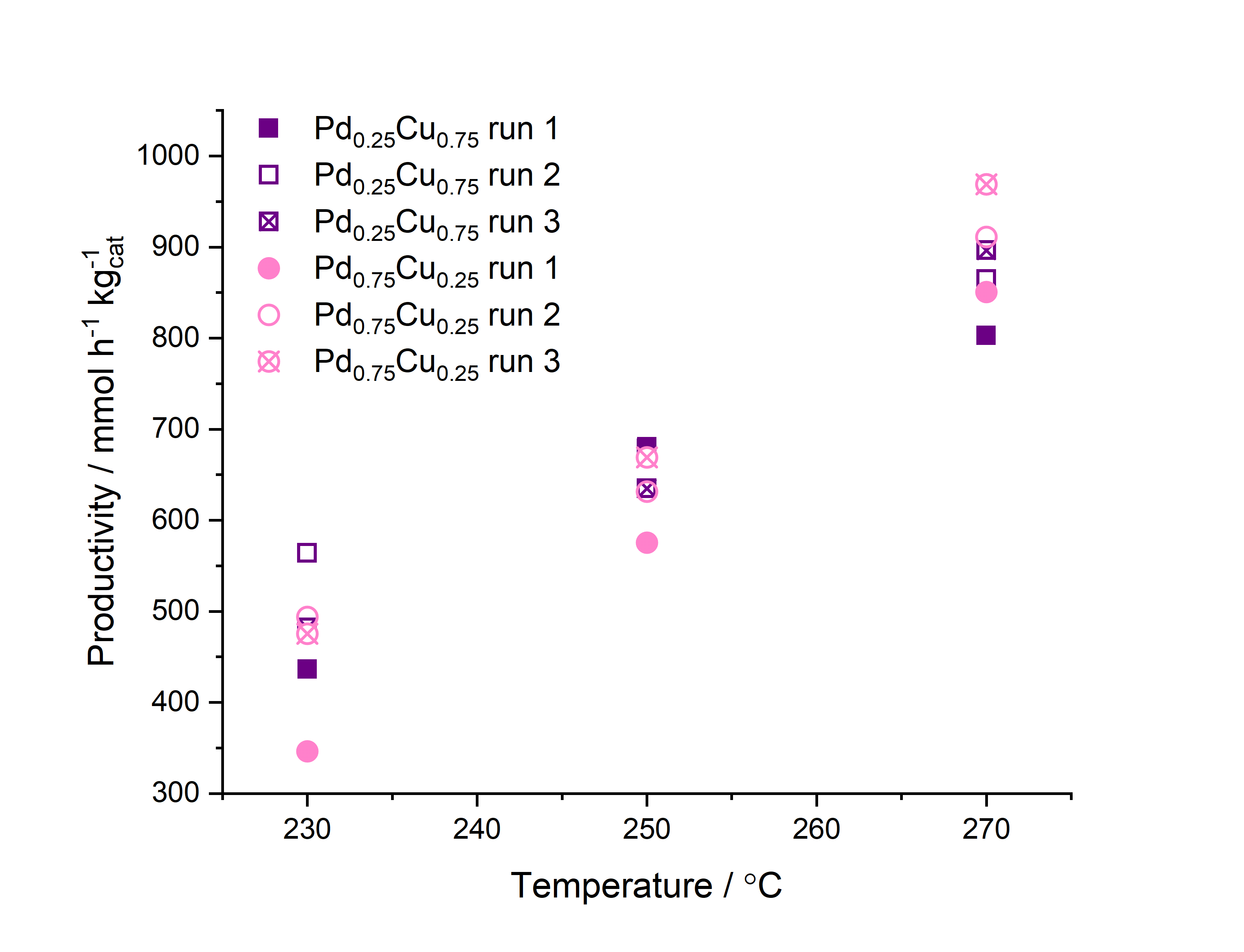


Figure S19. Methanol productivity for Pd_0.25_Cu_0.75_/ZnO and Pd_0.75_Cu_0.25_/ZnO on the first, second and third uses

**Supplementary note 6**

Following the initial reduction at 230 °C initial catalytic experiment (run 1), the catalyst was re-tested at 230, 250 and 270 °C without an additional reductive pre-treatment or any form of catalyst regeneration process. Following the second catalytic experiment (run 2), the cycle was repeated to obtain the data for run 3, again without any prior pre-treatment.

Table S3. Methanol productivity values for a range of PdCu catalysts reported in literature

| *Catalyst* | *Metal loading (wt.%)* | *T (°C)* | *Pressure (bar)* | *GHSV*  *(mL g^-1^_cat_ h^-1^)* | *MeOH productivity (mmol h^-1^ kg^-1^_cat_)* | *MeOH productivity (mol h^-1^ mol^-1^_metal_)* | *Reference* |
| --- | --- | --- | --- | --- | --- | --- | --- |
| **c**-PdCu/ZnO | 0.5 Pd & 0.5 Cu | 270 | 20 | 3600 | 1030 | 8.20 | **This work** |
| Pd(0.34)-Cu/SiO_2_ | 8.7 Pd & 10 Cu | 250 | 41 | 3600 | 1116 | 0.47 | 2 |
| Pd-Cu(0.25)/SiO_2_ | 5.7 Pd & 10 Cu | 250 | 50 | 30000 | 1630 | 0.77 | 3 |
| PdCu/ZrO_2_ | 5.7 Pd & 10 Cu | 250 | 41 | 3600 | 1872 | 0.89 | 4 |
| Pd-Cu(2.4)/SiO_2_ | 1.1 Pd & 1.3 Cu | 250 | 41 | 3600 | 233 | 0.76 | 5 |
| CuPd_0.1_/Al_2_O_3_ | 0.12 Pd & 10 Cu | 250 | 40 | 9000 | 620 | 0.39 | 6 |
|  |  |  |  | 72000 | 5910 | 3.73 |  |
| Cu-Pd/SiO_2_ | 0.8 Pd & 16.5 Cu | 280 | 40 | 4000 | 97 | 0.04 | 7 |
| 37.5PdCuZn/SiC | 39.4 Pd & 14.3 Cu & 46.3 Zn | 200 | 1^a^ | 7500 | 110 | 0.02^b^ | 8 |
| Pd-0.01-Cu/ZnO | 0.58 Pd & 47.3 Cu | 270 | 45 | 10800 | 6460 | 0.86 | 9 |
| Pd-Cu/ZnO/ZrO_2_ | 2 Pd & 36 Cu | 240 | 50 | 6000 | 7540 | 1.29 | 10 |
| 0.9Pd/CZA-zH | 0.94 Pd & 60 Cu | 180 | 28 | 2880 | 2496 | 0.24 | 11 |
| PdCu/PCN | 0.18 Pd & 0.16 Cu | 180 | 40 | - ^c^ | 286 | 6.8 | 12 |

a) 9:1 ratio of H_2_:CO_2_; b) calculated from Pd & Cu loadings only; c) Batch reactor

Table S4. Methanol productivity across the range of reaction temperatures for a commercially available Cu/ZnO/Al_2_O_3_ catalyst, along with Cu/ZnO and **s**-PdCu prepared by magnetron sputtering.

| *Catalyst* | *Metal loading (wt.%)* | *T (°C)* | *MeOH productivity (mol h^-1^ mol^-1^_metal_)* |
| --- | --- | --- | --- |
| Alfa Aesar CuO/ZnO/Al_2_O_3_^a^ | 50.7 Cu | 230 | 1.48 |
|  |  | 250 | 1.53 |
|  |  | 270 | 0.99 |
| Cu/ZnO | 1 Cu | 230 | 2.82 |
|  |  | 250 | 3.63 |
|  |  | 270 | 4.03 |
| **c**-PdCu/ZnO | 0.5 Pd & 0.5 Cu | 230 | 3.99 |
|  |  | 250 | 6.32 |
|  |  | 270 | 8.20 |

a) Catalyst composition reported as 10.1% Al_2_O_3_, 63.5 % CuO, 24.7% ZnO, 1.3 % MgO. Cu wt.% loading calculated to be 50.7 wt.% following *in situ* at 230 °C immediately before catalyst testing, assuming complete reduction of CuO to Cu.

**Supplementary References**

1 Davies, P. R. & Morgan, D. J. Practical guide for x-ray photoelectron spectroscopy: Applications to the study of catalysts. *Journal of Vacuum Science & Technology A* **38**, doi:10.1116/1.5140747 (2020).

2 Jiang, X., Koizumi, N., Guo, X. & Song, C. Bimetallic Pd–Cu catalysts for selective CO2 hydrogenation to methanol. *Applied Catalysis B: Environmental* **170-171**, 173-185, doi:<https://doi.org/10.1016/j.apcatb.2015.01.010> (2015).

3 Nie, X. *et al.* Mechanistic Understanding of Alloy Effect and Water Promotion for Pd-Cu Bimetallic Catalysts in CO2 Hydrogenation to Methanol. *ACS Catalysis* **8**, 4873-4892, doi:10.1021/acscatal.7b04150 (2018).

4 Lin, F. *et al.* Effects of supports on bimetallic Pd-Cu catalysts for CO2 hydrogenation to methanol. *Applied Catalysis A: General* **585**, 117210, doi:<https://doi.org/10.1016/j.apcata.2019.117210> (2019).

5 Jiang, X. *et al.* CO2 hydrogenation to methanol on PdCu bimetallic catalysts with lower metal loadings. *Catalysis Communications* **118**, 10-14, doi:<https://doi.org/10.1016/j.catcom.2018.09.006> (2019).

6 Pan, H. *et al.* Highly Efficient CuPd0.1/γ-Al2O3 Catalyst with Isolated Pd Species for CO2 Hydrogenation to Methanol. *ACS Sustainable Chemistry & Engineering* **11**, 7489-7499, doi:10.1021/acssuschemeng.3c00600 (2023).

7 Qiu, R. *et al.* CuPd bimetallic catalyst with high Cu/Pd ratio and its application in CO2 hydrogenation. *Applied Surface Science* **544**, 148974, doi:<https://doi.org/10.1016/j.apsusc.2021.148974> (2021).

8 Díez-Ramírez, J., Díaz, J. A., Sánchez, P. & Dorado, F. Optimization of the Pd/Cu ratio in Pd-Cu-Zn/SiC catalysts for the CO2 hydrogenation to methanol at atmospheric pressure. *Journal of CO2 Utilization* **22**, 71-80, doi:<https://doi.org/10.1016/j.jcou.2017.09.012> (2017).

9 Hu, B. *et al.* Hydrogen spillover enabled active Cu sites for methanol synthesis from CO2 hydrogenation over Pd doped CuZn catalysts. *Journal of Catalysis* **359**, 17-26, doi:<https://doi.org/10.1016/j.jcat.2017.12.029> (2018).

10 Shrivastaw, V. K. *et al.* Surface basicity induced Pd doped Cu/ZnO/ZrO2 for selective CO2 hydrogenation to Methanol. *Molecular Catalysis* **578**, 114966, doi:<https://doi.org/10.1016/j.mcat.2025.114966> (2025).

11 Fan, X. *et al.* Pd-modified CuO-ZnO-Al2O3 catalysts via mixed-phases-containing precursor for methanol synthesis from CO2 hydrogenation under mild conditions. *Carbon Resources Conversion* **7**, 100184, doi:<https://doi.org/10.1016/j.crcon.2023.05.003> (2024).

12 Wang, Y. *et al.* Ultralow-Content PdCu Disordered Nanoalloys on Carbon Nitrides for Unity-Selective CO2 Hydrogenation to Methanol. *Angewandte Chemie International Edition* **n/a**, e202508611, doi:<https://doi.org/10.1002/anie.202508611>.
